# Periodic Density Functional Theory Calculations of Uranyl Tetrachloride Compounds Engaged in Uranyl–Cation and Uranyl–Hydrogen Interactions: Electronic Structure, Vibrational, and Thermodynamic Analyses

**DOI:** 10.1021/acs.inorgchem.2c03476

**Published:** 2022-12-20

**Authors:** Logan J. Augustine, Harindu Rajapaksha, Mikaela Mary F. Pyrch, Maguire Kasperski, Tori Z. Forbes, Sara E. Mason

**Affiliations:** Department of Chemistry, University of Iowa, Iowa City, Iowa52242, United States

## Abstract

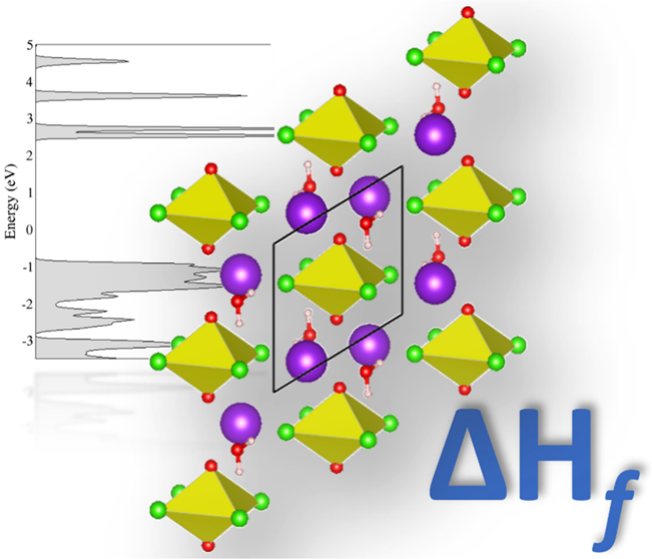

Solid-state uranyl hybrid structures are often formed through unique intermolecular interactions occurring between a molecular uranyl anion and a charge-balancing cation. In this work, solid-state structures of the uranyl tetrachloride anion engaged in uranyl–cation and uranyl–hydrogen interactions were studied using density functional theory (DFT). As most first-principles methods used for systems of this type focus primarily on the molecular structure, we present an extensive benchmarking study to understand the methods needed to accurately model the geometric properties of these systems. From there, the electronic and vibrational structures of the compounds were investigated through projected density of states and phonon analysis and compared to the experiment. Lastly, we present a DFT + thermodynamics approach to calculate the formation enthalpies (Δ*H*_f_) of these systems to directly relate to experimental values. Through this methodology, we were able to accurately capture trends observed in experimental results and saw good quantitative agreement in predicted Δ*H*_f_ compared to the value calculated through referencing each structure to its standard state. Overall, results from this work will be used for future combined experimental and computational studies on both uranyl and neptunyl hybrid structures to delineate how varying intermolecular interaction strengths relates to the overall values of Δ*H*_f_.

## Abstract 2

Solid-state uranyl tetrachloride compounds are investigated using density functional theory. We look to explore the role different non-covalent interactions (uranyl−cation and uranyl−hydrogen) may have on the systems’ electronic, vibrational, and thermodynamic properties. Reported is the use of a DFT + thermodynamics technique to calculate the formation enthalpies which are needed to make direct comparisons to the experiment.

## 1 Introduction

Uranium is shown to exist in a range of oxidation states from U(II) to U(VI),^1−3^ but in solid-state compounds the most common forms are U(IV) and U(VI). Under oxidizing conditions, the most stable oxidation state of uranium is U(VI), where it forms into the well-studied uranyl cation, [UO_2_]^2+^.^1,2,4^ Within this unit, the central U(VI) is strongly bound to two axial oxygen atoms at bond lengths between 1.70 and 1.80 Å with a O–U–O bond angle of ∼180^°^.^1,2,5^ Similar bonding is also observed for hexavalent neptunium and plutonium, but differences in their electronic structures lead to subtle changes in the overall bond strength.^6,7^ The substantial covalent bonding within the actinyl cation forces additional ligand interactions to primarily occur along the equatorial plane, often forming into square, pentagonal, and hexagonal bipyramidal geometries.^8^ The binding of these equatorial ligands leads to additional electron donation to the An(VI) metal center and weakening of the U=O_yl_ bond, an effect often observed through red-shifting of the ν_1_ and ν_3_ modes in Raman and IR spectroscopy.^9−11^ U=O_yl_ bond weakening is known to increase the Lewis basicity of the axial oxo groups, enhancing their ability to interact with Lewis acids through different non-covalent interactions (NCIs).^12^ These second-sphere NCIs, including Coulombic, hydrogen bonding, and halogen bonding, can influence the overall chemical and physical properties of the material,^12^ including structural topologies,^13^ redox potentials,^14,15^ and activation of vibrational modes.^16−18^

NCIs are mostly characterized in U(VI) systems, and Cahill and co-workers provide elegant examples of how these forces can be used to build unique solid-state supramolecular assemblies. Their systems utilize high concentrations of HCl to fully saturate the bonding along the uranyl equatorial plane and promote the formation of the uranyl tetrachloride anion, [UO_2_Cl_4_]^2–^, which is then crystallized using different charge-balancing organic heterocycles and molecules.^19−22^ As the equatorial plane is fully coordinated, the solid-state lattices within these systems are built primarily through extensive NCIs (*i.e.*, hydrogen bonding and halogen bonding) with the uranyl tetrachloride anion. While Coulombic forces are the drivers for solid-state assemblies, it is these NCIs which are responsible for determining the molecular level arrangement of [UO_2_Cl_4_]^2–^ solid phases.^19,22^ Furthermore, altered signals in uranyl absorption and emission spectroscopy for these compounds show that the NCIs have a direct influence on the electronic structure of the uranyl cation.^21^ However, the overall effect these interactions can have on different properties of the uranyl system and other related actinyl compounds is yet to be fully understood.

As the NCI network within uranyl hybrid materials can impact the optical properties of the materials, they are also likely to have impacts on their thermodynamics and overall formation energies (Δ*H*_f_). To the best of our knowledge, only one study by Cahill *et al.* evaluated the influence NCIs have on the value of Δ*H*_f_ for hybrid materials containing the [UO_2_Cl_4_]^2–^ anion.^23^ Results from this study showed a significant shift in Δ*H*_f_ for compounds with uranyl–cation interactions compared to those with uranyl–hydrogen interactions, but they were unable to correlate intermolecular interactions to changes in Δ*H*_f_ due to limited data and similarities in structural topologies within uranyl–hydrogen systems.^23^ This lack of experimental data prevents more in-depth analysis of the relationship between the stability of these structures and the strength of these NCIs within the solid-state lattice. Additionally, efforts will further be needed to understand related Np and Pu systems to provide added insights into the electronic structure of *f*-block elements. Only a handful of related Np compounds have been reported in the literature, but these systems display enhanced NCIs compared to their uranyl counterparts.^16,17,24,25^ However, the experimental determination of Δ*H*_f_ of neptunyl can be far more challenging than that of uranyl systems due to limited availability, high cost, and the increased radioactivity of neptunium-237.

The use of computational methods, such as density functional theory (DFT), to calculate the thermochemistry in these systems can assist in situations when experimental Δ*H*_f_ is difficult to calculate and would enable additional insights into the impacts of NCI on the Δ*H*_f_ of actinyl solid-state materials. Effort in this area requires additional benchmarking and development as the majority of *ab initio* studies performed on the uranyl and neptunyl structures are for purely molecular systems. To our knowledge, only one DFT study on a [UO_2_Cl_4_]^2–^ solid-state material has been reported in the literature.^26^ In the current work, we study the [UO_2_Cl_4_]^2–^ system in the assembled state through either uranyl–cation or uranyl–hydrogen NCIs, as there is experimental data to directly compare our results when it comes to calculating their formation enthalpies. Initially, we provide a benchmarking study for these systems to explore their geometric properties and then focus on their electronic, vibrational, and thermochemical properties. To calculate the enthalpies of formation, we present a DFT + thermodynamics framework, which combines total energy information from electronic structure calculations with experimental thermochemical data, as used in the study of a range of heterogeneous chemical reactions.^27−32^ Through this methodology, we obtain an agreement between the theoretical results and experimental data when investigating these uranyl solid-state systems, which is not observed when referencing structures to their atomic standard states.

## 2 Methodology

### 2.1 Computational Details

All periodic, spin-polarized DFT calculations were performed using the Vienna Ab initio Simulation Package (VASP).^33−35^ Both the generalized gradient approximation of Perdew–Burke–Ernzerhof (GGA-PBE)^36^ and a revised version for solids and surfaces (GGA-PBEsol)^37^ were used to model the exchange–correlation energy. All atoms were represented using projector augmented wavefunction pseudopotentials.^38,39^ Calculations used a 550 eV cutoff energy for the plane-wave basis set and gamma-centered Monkhorst–Pack *k*-grids^40^ with a mesh of at most 0.16 Å^–1^ spacing in the Brillouin zone. Full lists of *k*-grids are provided in Table S2. All structures were subjected to full geometry optimizations without symmetry constraints and converged to within 10 meV/Å in the forces and 1 × 10^–6^ eV in the total energy.

To form a basis of comparison with previous literature studies on uranyl molecular tetrachloride systems, we performed a series of benchmarking calculations to examine the accuracy and consistency of our employed methods to describe the properties of these systems. This included comparing both the GGA-PBE and GGA-PBEsol functionals, calculating with and without a Hubbard *U* correction,^41−43^ and employing two different van der Waals dispersion correction schemes (DFT-D3 of Grimme and DFT-D3 including the Becke-Johnson damping term).^44,45^ The Hubbard *U* correction was applied to the uranium *f* states following the approach of Dudarev *et al.,*(46) with a *U*–*J* value of 4.0 eV. This value was chosen based on the previous results of bulk systems involving U(VI) oxidation states.^26,47−49^

Vibrational frequencies for all systems were calculated through finite-displacement, as implemented in the Phonopy package.^50^ To avoid undesirable interactions between periodic repeat units upon atomic displacement, supercells, which were at least 10 Å in each lattice direction, were used for the structures. Results from these calculations were used for vibrational analysis and to calculate the zero-point vibrational energies for enthalpy corrections to the total DFT energy. All crystallographic figures were generated using the VESTA software.^51^

### 2.2 Structural Details

#### 2.2.1 Uranyl–Cation Structures

**Figure 1:**
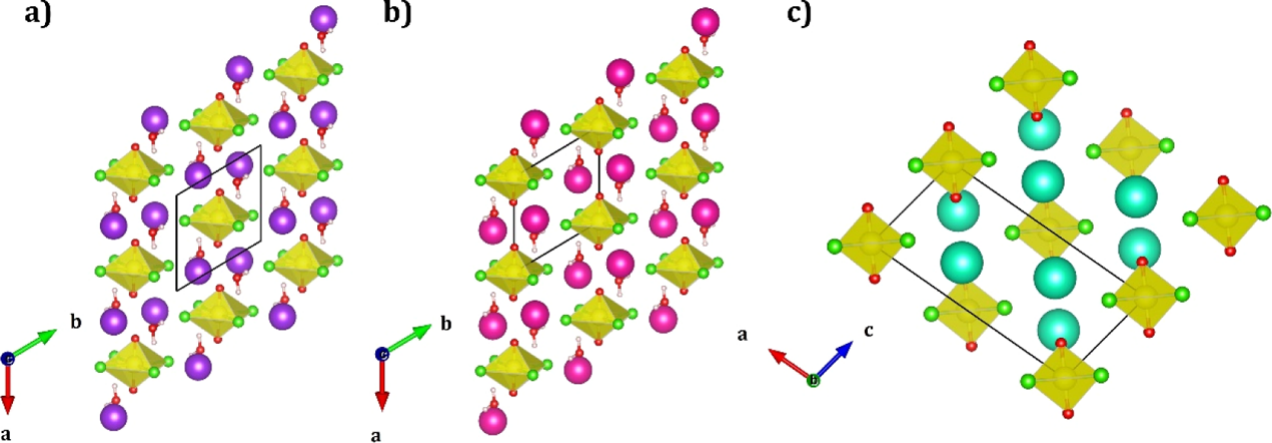
Crystal structures of the uranyl–cation systems: (a) K_2_[UO_2_Cl_4_]·2H_2_O **(1)**, (b) Rb_2_[UO_2_Cl_4_]·2H_2_O **(2)**, and (c) Cs_2_[UO_2_Cl_4_] **(3)**. Yellow polyhedrons are used to represent the uranium center. Red, green, white, purple, magenta, and cyan spheres represent oxygen, chloride, hydrogen, potassium, rubidium, and cesium atoms, respectively.

Figure 1a–c show the uranyl–cation structures studied in this work. Both K_2_[UO_2_Cl_4_]·2H_2_O **(1)** (Figure 1a) and Rb_2_[UO_2_Cl_4_]·2H_2_O **(2)** (Figure 1b) are isostructural and crystallize in the triclinic unit cell, *P*1̅. The structure of **(2)** has been reported previously in the literature,^7,52^ while **(1)** is a novel compound that is being reported herein (Table S1). Both structures contain one formula unit in the unit cell with the uranyl group having *C*_*i*_ site symmetry. Oxo groups are located trans to each other at distances of U=O_yl_ = 1.765 Å **(1)** and 1.773 Å **(2),** while chloride ligands are found along the equatorial plane at two distinct distances of U–Cl = 2.659 Å, 2.665 Å **(1)**, 2.665 and 2.669 Å **(2)**. Charge-balancing cations are located trans to each other along the same plane of the equatorial chloride atoms with unequal bifurcated K/Rb···Cl interactions at 3.236 Å, 3.279 Å **(1)** and 3.335 Å, 3.378 Å **(2)**, while also engaging in K/Rb···O_yl_ interactions above and below another uranyl group at 2.964 Å, 3.019 Å **(1)** and 3.019 Å, 3.059 Å **(2)**. The unit cells also contain two additional water molecules.

The structure of Cs_2_[UO_2_Cl_4_] **(3)** has been studied extensively both experimentally and theoretically.^7,53−55^ Unlike **(1)** and **(2)**, the structure of Cs_2_[UO_2_Cl_4_] crystallizes into a monoclinic unit cell, *C*2/*m*, containing two formula units in the lattice (Figure 1c). The uranyl group resides in a position with *C*_2h_ symmetry representing a pseudo-octahedral geometry. The U=O_yl_ bond distances are 1.776 Å with U–Cl distances of 2.671 Å. In the unit cell, the cesium atoms are located trans to each other, with one Cs atom slightly above and the other slightly below the chloride equatorial plane. These atoms have a bifurcated Cs···Cl interaction of 3.526 Å. The Cs atoms are also connected to another uranyl group as it lies directly above/below the oxo groups at Cs···O_yl_ = 3.275 Å.

#### 2.2.2 Uranyl–Hydrogen Structures

**Figure 2:**
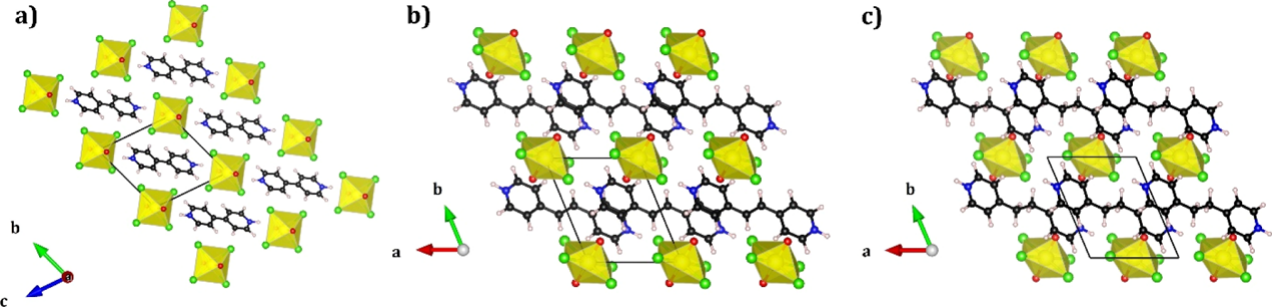
Crystal structures of the uranyl–hydrogen systems: (a) (C_10_H_10_N_2_)[UO_2_Cl_4_] **(4)**, (b) (C_12_H_12_N_2_)[UO_2_Cl_4_] **(5)**, and (c) (C_12_H_14_N_2_)[UO_2_Cl_4_] **(6)**. Yellow polyhedrons are used to represent the uranium center. Red, green, white, black, and blue spheres represent oxygen, chloride, hydrogen, carbon, and nitrogen atoms, respectively.

Each of the three uranyl–hydrogen systems studied here has been previously synthesized and characterized experimentally.^20^ All three structures in Figure 2a–c crystallize into the triclinic unit cell, *P*1̅, with a single formula unit in the lattice. In each of the systems, uranyl tetrachloride units are connected through a bifurcated hydrogen bonding interaction between the chloride atoms and the protonated amine group on the charge-balancing cation. The charge-balancing cations for the structures shown in Figure 2a–c are 4,4′-dipyridylamine **(4)**, trans-1,2-bis(4-pyridyl)ethylene **(5)**, and 1,2-bis(4-pyridyl)ethane **(6)**, respectively. U=O_yl_ bond distances in each system are 1.734 Å **(4)**, 1.763 Å **(5)**, and 1.762 Å **(6)**. Since the uranyl group is located at a site containing *C*_*i*_ symmetry, there are two distinct U–Cl bond lengths of 2.658 Å, 2.668 Å **(4);** 2.668 Å, 2.676 Å **(5)**; and 2.655 Å, 2.694 Å **(6)**. Compared to the uranyl–cation interactions seen in compounds **1–3**, the unequal bifurcated uranyl–hydrogen interactions in compounds **4–6** are much shorter with distances of Cl···H = 2.569 Å, 2.979 Å **(4)**; 2.645 Å, 2.642 Å **(5)**; 2.496 Å, 2.825 Å **(6).**

## 3 Results and Discussion

### 3.1 Structural Properties

Table 1 shows the calculated lattice parameters from this work using the PBE functional, a Hubbard *U* correction, and dispersion corrections with Becke-Johnson damping (PBE+*U*+D3BJ) in comparison to experimental values, while Table 2 displays the calculated intra- and inter-molecular interactions observed in each system. Benchmarking results for all eight methods were compared to the experiment and can be found in Tables S4–S12. As displayed in Table 1, the PBE + *U* + D3-BJ approach results in lattice parameter errors of less than two percent on all the uranyl systems studied, while all other approaches utilized resulted in errors of at least three percent.

**Table 1:** Calculated Lattice Parameters of the Uranyl–Cation and Uranyl–Hydrogen Crystal Structures (Using PBE+*U*+D3BJ) Compared to the Experiment^b^

uranyl–cation systems (compounds **1–3**)							
		*a*	*b*	*c*	α	β	γ
**(1)**	Exp.^a^	6.714	6.776	7.275	92.40	102.00	118.82
	Calc.	6.673 (−0.61%)	6.711 (−0.96%)	7.243 (−0.45%)	92.23 (−0.19%)	100.68 (−1.29%)	119.53 (+0.60%)
**(2)**	Exp.^7^	6.750	6.900	7.423	92.19	101.67	118.81
	Calc.	6.804 (+0.79%)	6.965 (+0.95%)	7.428 (+0.07%)	91.65 (−0.59%)	101.10 (−0.56%)	119.82 (+0.85%)
**(3)**	Exp.^7^	11.829	7.648	5.781	90	100.385	90
	Calc.	11.957 (+1.08%)	7.782 (+1.75%)	5.813 (+0.56%)	90	100.15 (−0.24%)	90

uranyl–hydrogen systems (compounds **4–6**)							
		*a*	*b*	*c*	α	β	γ
**(4)**	Exp.^20^	5.567	8.572	8.839	71.14	73.08	86.53
	Calc.	5.486 (−1.45%)	8.583 (+0.13%)	8.889 (+0.56%)	70.84 (−0.42%)	72.32 (−1.04%)	86.20 (−0.39%)
**(5)**	Exp.^20^	7.000	8.176	8.444	81.59	73.86	66.80
	Calc.	6.967 (−0.48%)	8.155 (−0.26%)	8.317 (−1.50%)	80.73 (−1.06%)	74.11 (+0.34%)	65.98 (−1.23%)
**(6)**	Exp.^20^	7.030	8.244	8.768	82.81	70.63	66.07
	Calc.	7.058 (+0.40%)	8.263 (0.24%)	8.600 (−1.91%)	81.85 (−1.15%)	70.54 (−0.12%)	65.71 (−0.55%)

a This work.

b All distances are reported in Å, and all angles are reported in degrees (°).

**Table 2:** Calculated Intra- and Inter-molecular Interactions of the Uranyl–Cation and Uranyl–Hydrogen Crystal Structures (Using PBE + *U* + D3-BJ) Compared to the Experiment^b^

uranyl–cation systems (compounds **1–3**)					
		U=O_yl_	U–Cl	M···Cl	M···O_yl_
**(1)**	Exp.^a^	1.765	2.659, 2.665	3.236, 3.279	2.964, 3.019
	Calc.	1.781	2.692, 2.694	3.231, 3.266	2.930, 2.991
**(2)**	Exp.^7^	1.773	2.665, 2.669	3.335, 3.378	3.019, 3.059
	Calc.	1.779	2.697, 2.699	3.353, 3.404	3.048, 3.089
**(3)**	Exp.^7^	1.776	2.671	3.526	3.275
	Calc.	1.778	2.704	3.559	3.332

uranyl–hydrogen systems (compounds **4–6**)				
		U=O_**yl**_	**U–Cl**	**Cl**···**H**
**(4)**	Exp.^20^	1.734	2.658, 2.668	2.569, 2.979
	Calc.	1.773	2.703, 2.708	2.354, 2.967
**(5)**	Exp.^20^	1.763	2.668, 2.676	2.642, 2.645
	Calc.	1.774	2.695, 2.712	2.473, 2.530
**(6)**	Exp.^20^	1.762	2.655, 2.694	2.496, 2.825
	Calc**.**	1.775	2.677, 2.734	2.204, 2.930

a This work.

b All distances are reported in Å.

To understand errors in calculated lattice parameters, we further focused on the performance of each method in calculating the intramolecular and intermolecular bonding for each system. Comparing the methods with and without a Hubbard *U* correction, we observe an increase in the U=O_yl_ bond distance by ∼0.04 Å when this correction term is not included in the calculation. This leads to a larger error compared to the experimental values, showing that the correction is needed to properly calculate the U=O_yl_ bond. A projected density of states (PDOS) analysis can be used to understand the interplay of Hubbard *U* and the U=O_yl_ bond (Figure S1). The valence states of uranyl tetrachloride are primarily composed of the chloride 2p orbitals. Not applying a Hubbard *U* correction results in increased uranium 5f electron density found in the same energy region. As covalency can be attributed to both orbital overlap and energy degeneracy,^56^ this suggests that there are increased covalent interactions between the uranium f and chloride p orbitals occurring along the equatorial plane of the uranyl unit, an effect also observed with U–Cl bond distances decreasing by ∼0.03–0.04 Å. As discussed *vide supra*, increased bonding and electron donation occurring along the equatorial plane often result in lengthening the U=O_yl_ bond. We conclude that the addition of the Hubbard *U* corrects for the over-delocalization of these uranium f orbitals and results in a more ionic U–Cl bonding interaction. Since weakening of the U=O_yl_ bond also increases the Lewis basicity of the oxo groups on the uranyl cation, the importance of applying a Hubbard *U* is also observed in the oxo-cation distances. This is seen through decreased oxo-cation distances in each of the systems without a *U* correction compared to their respective counterparts.

As expected, the use of dispersion corrections on uranyl hybrid materials had little effect on the calculated U=O_yl_ and U–Cl bond distances but decreased the M···O_yl_ and M···Cl distances. It was found that corrections which employed the Becke-Johnson damping term performed slightly better in calculated intermolecular distances, as shown previously in the literature.^45^ Differences between the PBE and PBEsol functionals were also small, with better performance from PBE in most systems. Based on these results, the rest of the work presented here uses the PBE+*U*+D3BJ methodology.

### 3.2 Electronic Structure

**Figure 3:**
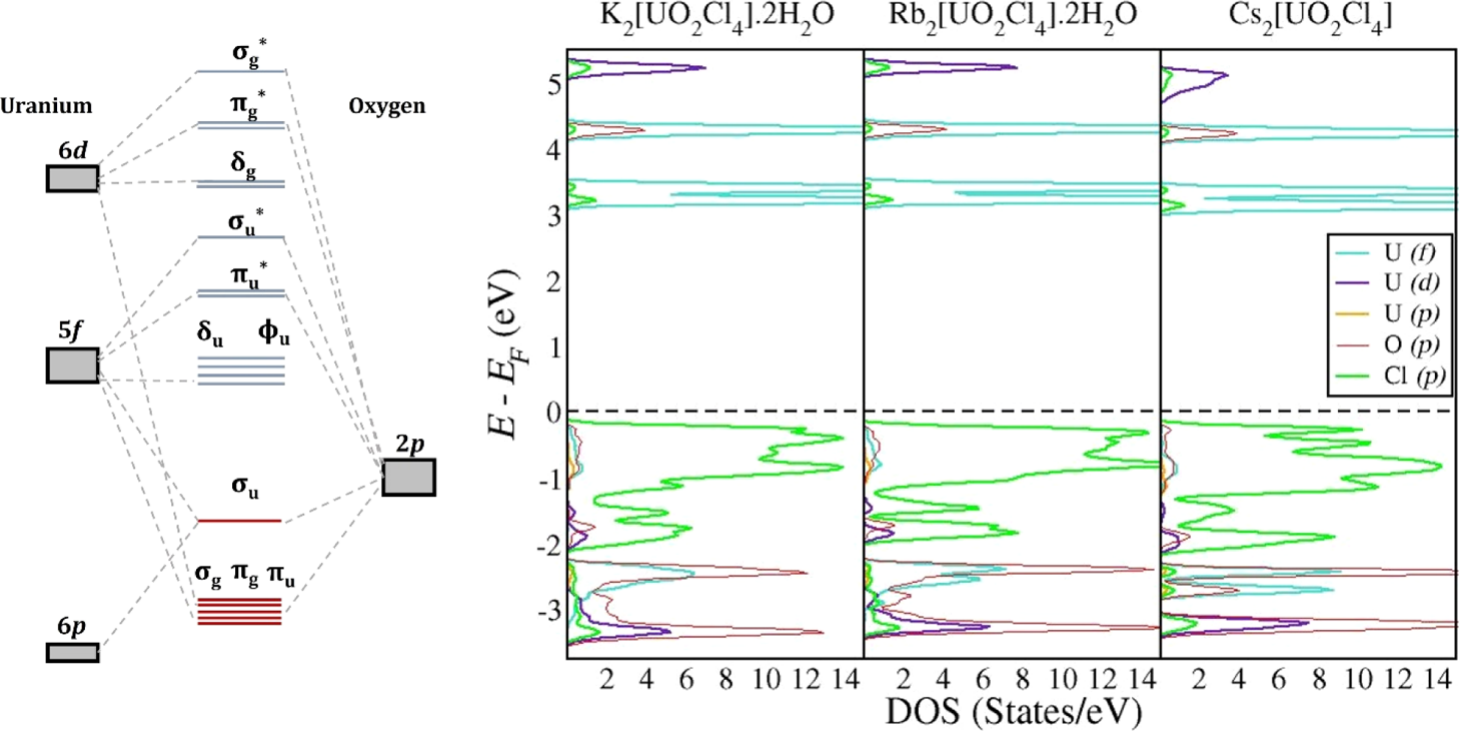
(Left) Representative molecular orbital diagram for the uranyl cation. Energy levels in red and blue represent occupied and unoccupied states, respectively. (Right) Calculated PDOS for the uranyl–cation structures.

The electronic structure of the uranyl cation [UO_2_]^2+^ has been characterized well both experimentally and theoretically.^53,54,57−59^Figure 3 shows the basic molecular orbital diagram of the uranyl unit in *D*_∞h_ symmetry, incorporating the most important valence and virtual orbitals. We also include the calculated PDOS for the uranyl–cation systems to make direct comparisons. To summarize the molecular orbital diagram of the uranyl unit: the lowest-lying valence orbitals are composed of mixing between uranium 5f_π_ and oxo 2p_π_ atomic orbitals, which are degenerate with the mixing of uranium 6d_π/σ_ and oxo 2p_π/σ_ atomic orbitals. The highest occupied molecular orbital in this system contains mixing of the uranium 5f_σ_ orbital with oxo 2p_σ_ atomic orbitals. This energy level is raised slightly due to a “pushing from below” effect from a small contribution of the uranium 6p_σ_ orbital in this level.^53,59^ The lowest unoccupied molecular orbital in the uranyl is composed of doubly degenerate uranium 5f_δ_ and 5f_ϕ_ nonbonding orbitals. This degeneracy tends to see a small split upon the inclusion of equatorial ligands. The remaining virtual orbitals consist of the antibonding 5f/6d and 2p orbitals along with the nonbonding 6d_δ_ orbitals on uranium.

Turning to the PDOS of the uranyl–cation systems, we observe similar profiles across the three systems (Figure 3). This shows that the corresponding charge-balancing alkali cation has little effect on the overall electronic structure of the uranyl system. Slightly below −3 eV, there is strong mixing occurring between the uranium 6d and oxo 2p orbitals, which correspond to the σ_g_ and π_g_ states displayed in the uranyl molecular orbital (MO) diagram. At −2.5 eV, there is strong mixing of the uranium 5f and oxo 2p orbitals, with a small amount of uranium 6p contribution, which corresponds well with the σ_u_ MO. The first unoccupied states (∼3.2 eV) in these systems consist of primarily uranium 5f orbitals, which can be associated with the nonbonding δ_u_ and ϕ_u_ MOs. These are followed by the antibonding 5f/2p states (∼4.2 eV) and the nonbonding 6d states (∼5.1 eV) in uranium. Also included in the plots are the chlorine 2p states, which are found primarily in the region from −2.0 to 0.0 eV. The energy levels of the equatorial ligands between the σ_u_ and nonbonding 5f orbitals match with what has been observed previously in both the experiment and theory. As discussed in Section 3.1, there is little mixing of the 2p chlorine state with the uranium 5f and 6d states, suggesting that the U–Cl equatorial interactions are primarily ionic in character.^10^ The overall PDOS for the uranyl–hydrogen systems follows that of the uranyl–cation systems (Figure S2), showing that both actinyl–cation interactions and actinyl–hydrogen interactions had little influence on the electronic energy levels of the main uranyl unit.

### 3.3 Vibrational Analysis

In *D*_∞h_ symmetry, the isolated uranyl cation displays four fundamental vibrational modes: the symmetric stretch (ν_1_), a doubly degenerate bending mode (ν_2_), and the antisymmetric stretch (ν_3_). Group theory analysis shows the ν_1_ mode is Raman active, while both ν_2_ and ν_3_ are IR active.^60^ In aqueous solution, the uranyl cation is expected to be coordinated by five water molecules around its equatorial plane, and the ν_1_ and ν_3_ modes can be observed as sharp bands at 872 and 965 cm^–1^ in Raman and IR spectroscopy, respectively.^61,62^ The engagement of the oxo group in NCIs can result in shifting of these vibrational energies or the activation of additional bands due to U=O_yl_ bond perturbation and weakening.^16,24,25^

Experimentally, optical scattering occurs near the Γ point in the Brillouin zone; therefore, we calculated the corresponding phonon eigenvalues and eigenvectors of the Hessian matrix at this point to investigate the uranyl–cation and uranyl–hydrogen vibrational spectra. As explained above, group theory can be used to determine the total number of IR and Raman active modes in each system,^63^ while the intensities of each mode can, in principle, be calculated using density functional perturbation theory (DFPT). In practice, DFPT with dispersion corrections is currently not implemented in VASP; therefore, IR and Raman intensities were not calculated in these structures. Also, the presence of the N-heterocyclic cations in the uranyl–hydrogen structures gives rise to vibrational modes in the range of 750–1000 cm^–1^, which overlap with the ν_1_ and ν_3_ modes we are particularly interested in, thus making their assignment based on group theory analysis alone more difficult. To overcome this, we use the approach described by Spano *et al.*,^47^ shown in eqs 1–3.
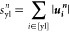
1
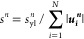
2
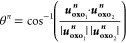
3Here, *s*_yl_^*n*^ represents the total displacement amplitude of the uranyl cation (*n*) for a particular normal mode, calculated by finding the norm (|***u***_***i***_^***n***^|) of the displacement vector for each atom in the cation [yl]. This is then normalized by the overall amplitude of the respective normal mode by summing over the displacement amplitudes for each atom (*N*) in the crystal structure (eq 2). eq 3 then represents the phase angle θ^*n*^ between the displacement vectors of both uranyl oxo groups (***u***_**oxo**_^***n***^). Using this approach allows us to identify vibrational modes which originate primarily from the motion of the uranyl cation (through larger values of *s*^*n*^) and based on the phase angle, can identify if the modes arise from symmetric (θ^*n*^ ∼ 180^°^) or asymmetric (θ^*n*^ ∼ 0^°^) stretching of the U=O_yl_ bonds.

#### 3.3.1 Raman Spectrum

Calculated values of the ν_1_ modes for each of the uranyl–cation and uranyl–hydrogen systems are shown in Table 3. Experimentally, the ν_1_ of **(1)** and **(2)** has been assigned at 839 cm^–1^. The calculated values for these systems are at 811 and 813 cm^–1^, showing a slight underestimation of the vibrational energy. The experimental and predicted values of the ν_1_ mode show that changing the charge-balancing cation between **(1)** and **(2)** has a negligible impact on the uranyl vibrational properties. This can be observed through similar U=O_yl_ bond distances in both systems. Therefore, the smaller M···O_yl_ distances found in **(1)** most likely arise from the smaller ionic size of the metal cation instead of stronger uranyl–cation interactions. The ν_1_ mode of **(3)** has been experimentally assigned at 832 cm^–1^, while our calculated mode at 806 cm^–1^ again underestimates the energy. **(3)** displays a similar U=O_yl_ bond to what was calculated for **(1)** and **(2)** but has a larger M···O_yl_ interaction distance. Therefore, the lower energy ν_1_ mode found in **(3)** likely arises from changes in solid-state packing in this system rather than any changes in the M···O_yl_ interaction strengths.

**Table 3:** Calculated ν_1_ and ν_3_ Normal Modes of Uranyl for the Uranyl–Cation and Uranyl–Hydrogen Structures^b^

uranyl structure						
vibrational mode	**(1)**	**(2)**	**(3)**	**(4)**	**(5)**	**(6)**
**ν**_**1**_**(cm**^**–1**^**)**	811 (839)^a^	813 (839)^7^	806 (832)^7^	825	821	818
**ν**_**3**_**(cm**^**–1**^**)**	961	965 (907)^7^	973 (922)^7^	977	987	981

a This work.

b Experimental values are shown in parentheses.

The symmetric stretching mode of **(4)** has been calculated to have an energy of 825 cm^–1^. Visualization of the phonon eigenvectors shows that there is additional motion of the bipyridine molecule in this mode (Figure S8). At 808 cm^–1^, there exists an additional Raman active mode produced by the combined motion of ν_1_ like stretching of the axial oxygen atoms and rocking of the nearest neighbor hydrogen on the bipyridine (Table S16 and Figure S8). The activation of combination modes similar to this has previously been observed in the neptunyl structure engaged in actinyl–hydrogen interactions with N-heterocyclic cations.^24^ The ν_1_ mode of **(5)** and **(6)** was calculated at 821 and 818 cm^–1^, respectively. Unlike **(4)**, there are no combination modes occurring in these structures, as visualization of the phonon eigenvectors shows atomic motion arising primarily from the uranyl or N-heterocyclic cation in this energy region. Comparing across the three uranyl–hydrogen systems, there is only a small variation (7 cm^–1^) in the symmetric stretch. This shows that any changes in intermolecular interactions played little role in the calculated mode energies, which can be expected as the amine groups interact more with the equatorial chloride ligand than the axial oxo atoms.

#### 3.3.2 IR Spectrum

The antisymmetric modes of **(1)**, **(2)**, and **(3)** were calculated at 961, 965, and 973 cm^–1^, respectively. Experimental values of **(2)** and **(3)** have been reported at 907 and 922 cm^–1^, showing that our method systematically overestimates the energy of this mode, the opposite of what was observed with the ν_1_ modes. Again, there is a small difference in the vibrational energies of **(1)** and **(2),** showing that any changes in uranyl–cation interactions have a negligible effect on the uranyl vibrational properties. The calculated ν_3_ of **(3)** is now higher in energy with respect to **(1)** and **(2)**, displaying an opposite trend from what was observed in the symmetric stretch. This further supports the idea that larger changes in vibrational energies for this system arise from differences in solid-state packing compared to any changes in uranyl–cation interaction strength.

We assign the ν_3_ modes of **(4)**, **(5)**, and **(6)** to 977, 987, and 981 cm^–1^, respectively. For structures **(4)** and **(5)**, the modes are found to be purely fundamental, while **(6)** shows a small splitting of the ν_3_ due to combination modes with the bipyridyl cation (Table S18 and Figure S10). Again, there is a small variation in the vibrational energy across the uranyl–hydrogen structures (10 cm^–1^), following what was observed with the ν_1_ modes, with differences in hydrogen interactions having little effect on the mode. We note that vibrational properties were also calculated for systems using the PBEsol functional as it has been found that this functional may be better suited for phonon calculations.^63−67^ Calculated modes were systematically higher in energy using the PBEsol versus PBE (Tables S13–S18). Since the ν_1_ mode was underestimated but ν_3_ was overestimated compared to the experiment, there is no clear improvement when using PBEsol to investigate the vibrational properties of the uranyl.

### 3.4 Formation Energies

Experimental formation enthalpies for uranyl–cation and uranyl–hydrogen systems have previously been calculated through isothermal acid solution calorimetry.^23^ Through this method, formation enthalpy reactions do not reference the system back to the standard states of each atom, but instead, values are calculated through the reaction performed to create each uranyl hybrid (Table 4). Because of this, a DFT + thermodynamics approach was used to calculate the formation enthalpies to match the reactions from experiment and directly relate their values. The utility of this approach arises from breaking the overall reaction into different categorical steps which are labeled Δ*H*_1_, Δ*H*_2_, and Δ*H*_3_. All energies in Δ*H*_1_ are calculated using DFT, while any aqueous or liquid component in the overall formation reaction is referenced as atomic species in their standard state in Δ*H*_1_ since their energetics can be difficult to calculate using plane-wave DFT. Calculated DFT total energy values are transformed to enthalpies by adding the vibrational zero-point energy correction into each structure. Δ*H*_2_ and Δ*H*_3_ incorporate tabulated experimental data to account for the energetics of the aqueous and liquid systems (HCl_(aq)_ and H_2_O_(l)_ in this work), which were referenced to their standard state in Δ*H*_1_. The standard state references in Δ*H*_1_, Δ*H*_2_, and Δ*H*_3_ cancel when added, yielding an overall formation reaction that aligns with the calorimetry experiments. An example of this approach for system **(1)** is provided in eqs 4–7 with the overall reaction formulas and energies for each system provided in the Supporting Information, S6.
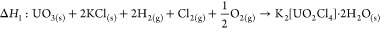
4
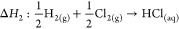
5
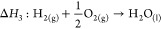
6
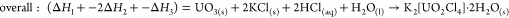
7

**Table 4:** Calculated Formation Enthalpies of the Uranyl–Cation and Uranyl–Hydrogen Structures through the DFT + Thermodynamics Framework

uranyl structure	formation enthalpy formula	calculated Δ*H*_f_(kJ/mol)	experimental Δ*H*_f_(kJ/mol)
**(1)**	UO_3(s)_ + 2KCl_(s)_ + 2HCl_(aq)_ + H_2_O_(l)_ → K_2_[UO_2_Cl_4_]·2H_2_O	–156.89	
**(2)**	UO_3(s)_ + 2RbCl_(s)_ + 2HCl_(aq)_ + H_2_O_(l)_ → Rb_2_[UO_2_Cl_4_]·2H_2_O	–167.63	
**(3)**	UO_3(s)_ + 2CsCl_(s)_ + 2HCl_(aq)_ → Cs_2_[UO_2_Cl_4_] + H_2_O_(l)_	–40.40	–44.28 ± 1.51
**(4)**	UO_3(s)_ + C_10_H_8_N_2(s)_ + 4HCl_(aq)_ → (C_10_H_10_N_2_)[UO_2_Cl_4_] + H_2_O_(l)_	–71.93	–94.28 ± 0.97
**(5)**	UO_3(s)_ + C_12_H_10_N_2(s)_ + 4HCl_(aq)_ → (C_10_H_10_N_2_)[UO_2_Cl_4_] + H_2_O_(l)_	–95.36	–116.45 ± 0.98
**(6)**	UO_3(s)_ + C_12_H_12_N_2(s)_ + 4HCl_(aq)_ → (C_12_H_14_N_2_)[UO_2_Cl_4_] + H_2_O_(l)_	–104.28	–125.23 ± 0.83

Table 4 shows the DFT-calculated formation enthalpies for each of the uranyl–cation and uranyl–hydrogen systems compared to their experimental values. Uranyl–hydrogen systems are systematically overestimated by +21 kJ/mol (∼0.21 eV), while **(3)** shows an error of +3.88 kJ/mol (∼0.04 eV). Comparing the uranyl–cation structures, we observe **(1)** and **(2)** having much more favorable formation enthalpies than **(3)**. This can be attributed to the increased number of intermolecular interactions in the unit cells of **(1)** and **(2)** due to the additional H_2_O molecules. Since **(1)** and **(2)** are isostructural, the only independent variables in the calculated formation enthalpies are the energetics of KCl/RbCl and K_2_[UO_2_Cl_4_]·2H_2_O/Rb_2_[UO_2_Cl_4_]·2H_2_O. Therefore, differences in Δ*H*_f_ arise either in the cohesive energies of KCl/RbCl or the interactions occurring in the corresponding uranyl solid-state systems. Comparison of calculated KCl and RbCl cohesive energies (Table S20) show that the KCl crystal has a stronger interaction energy by 14.81 kJ/mol. This is similar to the values of the overall differences in Δ*H*_f_ between **(1)** and **(2)**, showing that changes in Δ*H*_f_ arise primarily from the input energy required to break down the ionic crystal instead of increased stabilizing energy due to changes in intermolecular interactions between **(1)** and **(2)** through a differing metal cation.

Even though the error in calculated values of Δ*H*_f_ for (3) was larger compared to the uranyl–hydrogen systems, the overall trend in values follows what was calculated in the experiment. Experimentally, this trend was correlated to how easily the N-heterocyclic cation was protonated in solution, while no correlation was found between supramolecular interaction strengths (through bond distances) or the molar volumes of the crystal structure. Using the DFT + thermodynamics approach, we do not assess how protonation of the charge-balancing cation affects Δ*H*_f_, but similar to the reactions of **(1)** and **(2)**, each of the uranyl–hydrogen systems follows the same thermodynamic cycle. Therefore, the only independent variables are in the energetics of C_10_H_8_N_2_/C_12_H_10_N_2_/C_12_H_12_N_2_ and **(4)**/**(5)**/**(6)**. An opposite trend in the calculated cohesive energies of C_10_H_8_N_2_, C_12_H_10_N_2_, and C_12_H_12_N_2_ (Table S20) is observed compared to the uranyl–cation systems. Here, the cohesive energy of C_10_H_8_N_2_ is less than that of C_12_H_10_N_2_ and C_12_H_12_N_2_, but the overall Δ*H*_f_ of **(4)** is also higher than that of **(5)** and **(6)**. This may suggest that the uranyl–hydrogen interactions in **(5)** and **(6)** are stronger than those in **(4),** leading to more favorable formation enthalpies.

To understand the utility of the DFT + thermodynamics approach, the formation enthalpies were also calculated by referencing the atoms in each structure back to their standard states (Table S19). As expected, the calculated standard formation energies show significant errors compared to the experimental values as they are referenced to different equations. More importantly, although, the trends in Δ*H*_f_ shown in the experiment are no longer captured when referencing each system back to its standard state. This can be observed through structure **(3)** now having a more favorable formation enthalpy compared to the uranyl–hydrogen systems, and the uranyl–hydrogen systems now follow **(5)** < **(4)** < **(6)**.

## 4 Conclusions

In this work, solid-state periodic DFT calculations were performed on the uranyl tetrachloride engaged in uranyl–cation and uranyl–hydrogen interactions. Benchmarking studies of different exchange–correlation functionals and dispersion corrections were carried out on the systems, showing that the PBE+*U*+D3BJ methodology reproduced geometric and electronic structure parameters most accurately. It was found that the addition of a Hubbard *U* correction was most important for accurately describing the bonding interactions in the hybrid structures, while the functional (PBE/PBEsol) and dispersion correction (D3-Grimme/D3-BJ) types were less significant. Additionally, the electronic and vibrational properties of the uranyl were studied through PDOS and phonon analysis. Changing the intermolecular interactions through differing alkali or N-heterocyclic cations had little effect on the electronic and vibrational energy levels of these systems. The vibrational calculations did show the appearance of combination modes between uranyl and the N-heterocyclic cation in both Raman and IR spectroscopy, which is an important consideration when assigning vibrational bands within these systems.

Lastly, we adapted a DFT + thermodynamics framework to calculate the formation enthalpies of each structure. Using this approach, we were able to predict values of Δ*H*_f_ to within ∼21 kJ/mol compared to the experiment for the uranyl–hydrogen systems, while (4) only showed an error of ∼4 kJ/mol. More importantly, the method was able to accurately capture the experimental trends in Δ*H*_f_ observed between each system, contrary to referencing each system back to its standard state. This showed the utility and need of the DFT + thermodynamics approach when studying these reactions. Future work in this area will further focus on understanding how changes in actinyl intermolecular interaction strength may affect the formation energies of these hybrid materials. We present a way to interpret changes in their strength through the calculation of cohesive energies in reactant systems, as the current thermodynamic cycle does not separate out these terms for quantitative measurement. Therefore, work is currently being performed both experimentally and computationally on the uranyl and neptunyl hybrid materials, engaging NCIs to increase our understanding in this area.

## Supporting Information Available

Synthesis of K_2_[UO_2_Cl_4_]·2H_2_O; crystal structure information of K_2_[UO_2_Cl_4_]·2H_2_O; additional computational details and DFT benchmarking results; electronic structure; vibrational analysis; formation enthalpies; cohesive energies; and DFT-optimized structures (PDF)
