# Supplementary material for: Periodic Density Functional Theory Calculations of Uranyl Tetrachloride Compounds Engaged in Uranyl–Cation and Uranyl–Hydrogen Interactions: Electronic Structure, Vibrational, and Thermodynamic Analyses
Source: Inorg Chem. 2022 Dec 20;62(1):372–80. doi: 10.1021/acs.inorgchem.2c03476 (PMC9832540; doi:10.1021/acs.inorgchem.2c03476)
Supplement: Supplementary file 1 — ic2c03476_si_001.pdf [file ic2c03476_si_001.pdf]

Supporting Information

Periodic Density Functional Theory Calculations of Uranyl Tetrachloride Compounds Engaged in Uranyl-Cation and Uranyl-Hydrogen Interactions: Electronic Structure, Vibrational, and Thermodynamic Analysis

Logan J. Augustine,[a] Harindu Rajapaksha,[a] Mikaela Mary F. Pyrch,[a] Maguire Kasperski,[a] Tori Z. Forbes,[a] Sara E. Mason[a,*]

[a]University of Iowa, Department of Chemistry, Iowa City, IA 52242, USA

[*]Corresponding Author: Sara E. Mason

Table of Contents

S1. Synthesis of $K_2[UO_2Cl_4]$□$2H_2O$ 4

S2. Crystal structure information of $K_2[UO_2Cl_4]$□$2H_2O$ 4

Table S1: Crystallographic information of $K_2[UO_2Cl_4]\cdot 2H_2O$ system. 4

S3. Additional Computational Details and DFT benchmarking results 5

Table S2. Monkhorst-Pack *k*-grids used in optimization of each uranyl-hybrid structure 5

Table S3. Computational details on reactant systems used for thermodynamic and cohesive energy calculations. References are provided for selected systems for their associated crystal structures/experimental cif files. 5

Table S4: Comparison between the calculated and experimental unit cell parameters of $K_2[UO_2Cl_4]\cdot 2H_2O$ system. All distances are reported in Angstroms (Å), while angles reported in degrees (°). 7

Table S5: Comparison between the calculated and experimental bond and interaction distances of $K_2[UO_2Cl_4]\cdot 2H_2O$ system. All distances are reported in Angstroms (Å). 7

Table S6: Comparison between the calculated and experimental unit cell parameters of $Rb_2[UO_2Cl_4]\cdot 2H_2O$ system. All distances are reported in Angstroms (Å), while angles reported in degrees (°). 8

Table S7: Comparison between the calculated and experimental bond and interaction distances of $Rb_2[UO_2Cl_4]\cdot 2H_2O$ system. All distances are reported in Angstroms (Å). 8

Table S8: Comparison between the calculated and experimental unit cell parameters of $Cs_2[UO_2Cl_4]$ system. All distances are reported in Angstroms (Å), while angles reported in degrees (°). 9

Table S9: Comparison between the calculated and experimental bond and interaction distances of $Cs_2[UO_2Cl_4]$ system. All distances are reported in Angstroms (Å), while angles reported in degrees (°). 10

Table S10: Comparison between the calculated and experimental unit cell parameter, bond and interaction distances of $C_{10}H_{10}N_2[UO_2Cl_4]$ system. All distances are reported in Angstroms (Å), while angles reported in degrees (°). 11

Table S11: Comparison between the calculated and experimental unit cell parameter, bond and interaction distances of $C_{12}H_{12}N_2[UO_2Cl_4]$ system. All distances are reported in Angstroms (Å), while angles reported in degrees (°). 11

Table S12: Comparison between the calculated and experimental unit cell parameter, bond and interaction distances of $C_{12}H_{14}N_2[UO_2Cl_4]$ system. All distances are reported in Angstroms (Å), while angles reported in degrees (°). 12

S4. Electronic structure 13

Figure S1: Calculated projected density of states for the uranyl-cation structures without (left) and with (right) a Hubbard *U* correction. 13

Figure S2. Calculated projected density of states for the uranyl-hydrogen structure 14

S5. Vibrational analysis 15

S5.1 Experimental data 15

Figure S3: Fitted experimental Raman data of the powder $K_2[UO_2Cl_4]\cdot 2H_2O$ 15

S5.2 Calculated phonon frequencies, uranyl displacement amplitudes, and oxo displacement phase angle 15

Table S13: Vibrational analysis of each phonon mode of $K_2[UO_2Cl_4]$. Modes corresponding to the symmetric (ν_1) and asymmetric (ν_3) stretch of the uranyl cation are highlighted in red. 16

Table S14: Vibrational analysis of each phonon mode of $Rb_2[UO_2Cl_4].2H_2O$. Modes corresponding to the symmetric (ν_1) and asymmetric (ν_3) stretch of the uranyl cation are highlighted in red. 17

Table S15: Vibrational analysis of each phonon mode of $Cs_2[UO_2Cl_4]$. Modes corresponding to the symmetric (ν_1) and asymmetric (ν_3) stretch of the uranyl cation are highlighted in red. 19

Table S16: Vibrational analysis of each phonon mode of $C_{10}H_{10}N_2[UO_2Cl_4]$. Modes corresponding to the symmetric (ν_1) and asymmetric (ν_3) stretch of the uranyl cation, along with combination modes of the N-heterocyclic cation are highlighted in red. 21

Table S17: Vibrational analysis of each phonon mode of $C_{12}H_{12}N_2[UO_2Cl_4]$. Modes corresponding to the symmetric (ν_1) and asymmetric (ν_3) stretch of the uranyl cation, along with combination modes of the N-heterocyclic cation are highlighted in red. 23

Table S18: Vibrational analysis of each phonon mode of $C_{12}H_{14}N_2[UO_2Cl_4]$. Modes corresponding to the symmetric (ν_1) and asymmetric (ν_3) stretch of the uranyl cation, along with combination modes of the N-heterocyclic cation are highlighted in red. 25

Figure S4. Calculated phonon densities of states for each of the uranyl-cation and uranyl-hydrogen structures 28

S5.3 Eigenvector projections of the calculated phonon modes. 28

Figure S5: Phonon eigenvectors of the symmetric (ν_1) (left) and asymmetric (ν_3) (right) stretch of the uranyl cation in $K_2[UO_2Cl_4].2H_2O$. Figures A and B correspond PBE+*U*+BJ, while Figures C and D correspond to PBEsol+*U*+BJ. 28

Figure S6: Phonon eigenvectors of the symmetric (ν_1) (left) and asymmetric (ν_3) (right) stretch of the uranyl cation in $Rb_2[UO_2Cl_4].2H_2O$. Figures A and B correspond PBE+*U*+BJ, while Figures C and D correspond to PBEsol+*U*+BJ. 29

Figure S7: Phonon eigenvectors of the symmetric (ν_1) (left) and asymmetric (ν_3) (right) stretch of the uranyl cation in $Cs_2[UO_2Cl_4]$. Figures A-D correspond PBE+*U*+BJ, while Figures E-H correspond to PBEsol+*U*+BJ. 30

Figure S8: Phonon eigenvectors of the symmetric (ν_1), asymmetric (ν_3), and combination modes observed in $C_{10}H_{10}N_2[UO_2Cl_4]$. (Highlighted red in Table S14). Figures A-C correspond PBE+*U*+BJ, while Figures D-F correspond to PBEsol+*U*+BJ. 31

Figure S9: Phonon eigenvectors of the symmetric (ν_1), asymmetric (ν_3), and combination modes observed in $C_{12}H_{12}N_2[UO_2Cl_4]$. (Highlighted red in Table S15). Figures A-B correspond PBE+*U*+BJ, while Figures C-E correspond to PBEsol+*U*+BJ. 32

Figure S10: Phonon eigenvectors of the symmetric (ν_1), asymmetric (ν_3), and combination modes observed in $C_{12}H_{14}N_2[UO_2Cl_4]$. (Highlighted red in Table S14). Figures A-C correspond PBE+*U*+BJ, while Figures D-F correspond to PBEsol+*U*+BJ. 33

S6. Formation Enthalpies 34

S6.1 DFT + Thermodynamics 34

S6.2 Formation Enthalpies Referenced to Standard States 35

Table S19: Calculated formation enthalpies of the uranyl-cation and uranyl-hydrogen structures as referenced to the standard state of each atom. 35

S7. Cohesive Energies 36

Table S20. Calculated cohesive energies for the ionic and organic reactant crystals. 36

S8. DFT Optimized Structures 37

S9. References 42

S1. Synthesis of $K_2[UO_2Cl_4]$□$2H_2O$

Synthesis Caution! The uranyl acetate di-hydrate ($UO_2(CH_3COO)_2$. $2H_2O$) contain depleted uranium, standard precautions for handling radioactive and toxic substances should be followed.

$UO_2(CH_3COO)_2$. $2H_2O$ (International Bio-Analytical Industries, 98-102%, 0.218 g) was dissolved in 6 mL of Methanol and 1 mL conc HCl (12 N) by heating at 85 °C with constant stirring for ~10 minutes. 1.5 mL of this solution was mixed with 0.01 g of KCl (Aldrich, ≥99%) in a scintillation vial and left to slow evaporate at room temperature. Over the course of several days, single crystals for single crystal X-ray diffraction were harvested for the mother liquor.

S2. Crystal structure information of $K_2[UO_2Cl_4]$□$2H_2O$

Table S1: Crystallographic information of $K_2[UO_2Cl_4]\cdot 2H_2O$ system.

**Empirical Formula**	$K_2UO_4Cl_4$	**Θ range (°)**	5.804 to 50/7
**Formula weight**	526.06	**Limiting indices**	$-8 \le h \le 8$
**Space group**	P-1		$-7 \le k \le 8$
**a (Å)**	6.7144(3)		$-8 \le l \le 8$
**b (Å)**	6.7764(3)	**Ref. collected/unique**	12650
**c(Å)**	7.2755(4)	**R_{int}**	0.0766
**α (°)**	92.402(2)	**Data/restraints/parameters**	1029/0/53
**β (°)**	101.994(2)	**GOF on F^2**	1.135
**γ (°)**	118.821(2)	**Final R indices**	$R_1 = 0.0245$
**V($Å^3$)**	279.78(2)	**[I>2σ(I)]**	$wR_2 = 0.0649$
**Z**	1	**R indices (all data)**	$R_1 = 0.0245$
**P (g/cm^3)**	3.122		$wR_2 = 0.0649$
**μ (mm^{-1})**	16.172	**Largest peak and hole** ($Å^{-3}$)	1.65 and -1.74
**F (000)**	234.0		

S3. Additional Computational Details and DFT benchmarking results

Table S2. Monkhorst-Pack *k*-grids used in optimization of each uranyl-hybrid structure

Structure	(1)	(2)	(3)	(4)	(5)	(6)
***k*-grid**	6×6×6	6×6×6	4×6×6	8×6×6	6×6×6	6×6×6

Table S3. Computational details on reactant systems used for thermodynamic and cohesive energy calculations. References are provided for selected systems for their associated crystal structures/experimental cif files.

Structure	Lattice Parameters (Å)	# of atoms in unit cell	*k*-grid
$UO_{3(s)}$[1]	a=b=3.826 c=4.165<br>α=β=90° γ=120°	4	6×6×6
$KCl_{(s)}$	a=b=c = 6.271<br>α=β=γ = 90°	8	10×10×10
$RbCl_{(s)}$	a=b=c = 6.590<br>α=β=γ = 90°	8	10×10×10
$CsCl_{(s)}$	a=b=c = 4.136<br>α=β=γ = 90°	2	10×10×10
$C_{10}H_8N_{2(s)}$[2]	a=8.609 b=8.507 c=10.922<br>α=84.62° β=84.50° γ=79.64°	80	6×6×4
$C_{12}H_{10}N_{2(s)}$[a]	a=5.680 b=10.481 c=7.422<br>α=90° β=91.60° γ=90°	48	8×6×6
$C_{12}H_{12}N_{2(s)}$[3]	a=5.446 b=7.799 c=11.115<br>α=90° β=98.65° γ=90°	52	8×6×6
$U_{(s)}$	a=3.377 b=5.852 c=5.633<br>α=β=γ = 90°	4	16×12×12
$K_{(s)}$	a=b=c = 4.469<br>α=β=γ = 109.47°	1	18×18×18
$Rb_{(s)}$	a=b=c = 4.810<br>α=β=γ = 109.47°	1	18×18×18
$Cs_{(s)}$	a=b=c = 5.229<br>α=β=γ = 109.47°	1	18×18×18

**$C_{(s)}$**	a=b=2.466 c=6.706<br>α=β=90° γ=120°	4	15×15×8
**$H_2O_{(g)}$**	a=b=c = 25.000<br>α=β=γ = 90°	3	1×1×1
**$H_{2(g)}$**	a=b=c = 25.000<br>α=β=γ = 90°	2	1×1×1
**$N_{2(g)}$**	a=b=c = 25.000<br>α=β=γ = 90°	2	1×1×1
**$O_{2(g)}$**	a=b=c = 25.000<br>α=β=γ = 90°	2	1×1×1
**$Cl_{2(g)}$**	a=b=c = 25.000<br>α=β=γ = 90°	2	1×1×1
**$C_{10}H_8N_{2(g)}$**	a=b=c = 30.000<br>α=β=γ = 90°	20	1×1×1
**$C_{12}H_{10}N_{2(g)}$**	a=b=c = 30.000<br>α=β=γ = 90°	24	1×1×1
**$C_{12}H_{12}N_{2(g)}$**	a=b=c = 30.000<br>α=β=γ = 90°	26	1×1×1
**$K_{(g)}$**	a=b=c = 25.000<br>α=β=γ = 90°	1	1×1×1
**$Rb_{(g)}$**	a=b=c = 25.000<br>α=β=γ = 90°	1	1×1×1
**$Cs_{(g)}$**	a=b=c = 25.000<br>α=β=γ = 90°	1	1×1×1
**$Cl_{(g)}$**	a=b=c = 25.000<br>α=β=γ = 90°	1	1×1×1

[a]CCDC deposition number: 1828730

Table S4: Comparison between the calculated and experimental unit cell parameters of $K_2[UO_2Cl_4]{\cdot}2H_2O$ system. All distances are reported in Angstroms (Å), while angles reported in degrees (°).

$K_2[UO_2Cl_4]{\cdot}2H_2O$	***a***	***b***	***c***	***α***	***β***	***γ***
**Experimental[a]**	6.714	6.776	7.275	92.40	102.00	118.82
**PBE**	7.031 (+4.72%)	6.831 (+0.81%)	7.283 (+0.10%)	91.16 (-1.34%)	100.18 (-1.79%)	119.62 (+0.67%)
**PBE+*U***	6.913 (+2.95%)	6.856 (+1.17%)	7.383 (+1.47%)	91.79 (-0.66%)	100.87 (-1.10%)	119.02 (+0.16%)
**PBE+D3-Grimme**	6.861 (+2.19%)	6.786 (+0.14%)	7.213 (-0.85%)	91.42 (-1.05%)	100.61 (-1.36%)	120.45 (+1.37%)
**PBE+D3-BJ**	6.762 (+0.72%)	6.729 (-0.69%)	7.151 (-1.71%)	91.70 (-0.76%)	100.69 (-1.28%)	120.13 (+1.10%)
**PBE+*U*+D3-Grimme**	6.759 (+0.66%)	6.778 (+0.02%)	7.320 (+0.61%)	92.03 (-0.41%)	100.62 (-1.35%)	119.89 (+0.90%)
**PBE+*U*+D3-BJ**	6.673 (-0.61%)	6.711 (-0.96%)	7.243 (-0.45%)	92.22 (-0.19%)	100.68 (-1.29%)	119.53 (+0.59%)
**PBEsol+*U*+D3-Grimme**	6.580 (-2.00%)	6.605 (-2.52%)	7.155 (-1.64%)	92.59 (+0.21%)	100.27 (-1.72%)	119.80 (+0.82%)
**PBEsol+*U*+D3-BJ**	6.504 (-3.13%)	6.584 (-2.82%)	7.099 (-2.43%)	92.69 (+0.32%)	100.63 (-1.34%)	119.65 (+0.69%)

[a]This work

Table S5: Comparison between the calculated and experimental bond and interaction distances of $K_2[UO_2Cl_4]{\cdot}2H_2O$ system. All distances are reported in Angstroms (Å).

$K_2[UO_2Cl_4]{\cdot}2H_2O$	**U=O$_{yl}$**	**U-Cl**	***M***···**Cl**	***M***···**O$_{yl}$**
**Experimental[a]**	1.765	2.659, 2.665	3.236, 3.279	2.964, 3.019
**PBE**	1.820	2.665, 2.670	3.345, 3.395	2.893, 3.024
**PBE+*U***	1.782	2.703, 2.710	3.286, 3.389	2.990, 3.157
**PBE+D3-Grimme**	1.817	2.657, 2.660	3.314, 3.318	2.890, 2.960
**PBE+D3-BJ**	1.817	2.653, 2.658	3.259, 3.274	2.849, 2.914
**PBE+*U*+D3-Grimme**	1.781	2696, 2.697	3.273, 3.325	2.982, 3.048
**PBE+*U*+D3-BJ**	1.781	2.692, 2.694	3.231, 3.266	2.930, 2.991
**PBEsol+*U*+D3-Grimme**	1.771	2.665, 2.667	3.532	2.887, 2.886
**PBEsol+*U*+D3-BJ**	1.772	2.660, 2.665	3.481,	2.846

[a]This work

Table S6: Comparison between the calculated and experimental unit cell parameters of $Rb_2[UO_2Cl_4]·2H_2O$ system. All distances are reported in Angstroms (Å), while angles reported in degrees (°).

$Rb_2[UO_2Cl_4]·2H_2O$	*a*	*b*	*c*	*α*	*β*	*γ*
**Experimental[4]**	6.795(5)	6.929(5)	7.457(4)	91.96(5)	102.13(5)	118.82(6)
**PBE**	7.125 (+4.85%)	7.111 (+2.63%)	7.479 (+0.29%)	90.60 (-1.48%)	100.87 (-1.24%)	119.95 (+0.95%)
**PBE+*U***	7.017 (+3.27%)	7.123 (+2.79%)	7.568 (+1.49%)	91.23 (-0.79%)	101.05 (-1.05%)	119.52 (+0.58%)
**PBE+D3-Grimme**	7.000 (+3.01%)	7.046 (+1.68%)	7.393 (-0.86%)	90.88 (-1.17%)	101.24 (-0.87%)	120.49 (+1.40%)
**PBE+D3-BJ**	6.909 (+1.68%)	6.966 (+0.54%)	7.326 (-1.75%)	91.05 (-0.99%)	101.10 (-1.01%)	120.31 (+1.25%)
**PBE+*U*+D3-Grimme**	6.892 (+1.43%)	6.970 (+0.59%)	7.525 (0.91%)	91.52 (-0.48%)	100.63 (-1.47%)	119.68 (+0.72%)
**PBE+*U*+D3-BJ**	6.804 (+0.13%)	6.965 (+0.51%)	7.428 (-0.39%)	91.651 (-0.33%)	101.10 (-1.00%)	119.82 (+0.72%)
**PBEsol *U*+D3-Grimme**	6.695 (+0.81%)	6.716 (-2.65%)	7.377 (-0.61%)	92.12 (-0.07%)	99.78 (-1.86%)	119.17 (+0.30%)
**PBEsol *U*+D3-BJ**	6.612 (-1.95%)	6.773 (-1.83%)	7.280 (-1.92)	92.37 (+0.19%)	100.71 (-0.94%)	119.71 (+0.30%)

Table S7: Comparison between the calculated and experimental bond and interaction distances of $Rb_2[UO_2Cl_4]·2H_2O$ system. All distances are reported in Angstroms (Å).

$Rb_2[UO_2Cl_4]·2H_2O$	$U{=}O_{yl}$	U-Cl	$M\cdots Cl$	$M\cdots O_{yl}$
**Experimental[4]**	1.773	2.665, 2.669	3.335, 3.378	3.019, 3.059
**PBE**	1.812	2.669, 2.675	3.457, 3.514	3.038, 3.113
**PBE+*U***	1.781	2.707, 2.713	3.412, 3.513	3.128, 3.207
**PBE+D3-Grimme**	1.815	2.660, 2.663	3.383, 3.398	2.975, 3.022
**PBE+D3-BJ**	1.816	2.664, 2.664	3.433, 3.459	3.025, 3.084
**PBE+*U*+D3-Grimme**	1.780	2.701, 2.701	3.401, 3.469	3.097, 3.153
**PBE+*U*+D3-BJ**	1.779	2.697, 2.699	3.353, 3.404	3.048, 3.089
**PBEsol+*U*+D3-Grimme**	1.770	2.671, 2669	3.365, 3.347	2.992, 3.048
**PBEsol+*U*+D3-BJ**	1.770	2.666, 2.669	3.281, 3.212	2.951, 2.987

Table S8: Comparison between the calculated and experimental unit cell parameters of $Cs_2[UO_2Cl_4]$ system. All distances are reported in Angstroms (Å), while angles reported in degrees (°).

$Cs_2[UO_2Cl_4]$	*A*	*b*	*c*	*α*	*Β*	*γ*

**Experimental[4]**	11.829(5)	7.648(3)	5.781(2)	90	100.385(4)	90
**PBE**	12.260 (+3.64%)	7.843 (2.56%)	5.978 (+3.41%)	90 (0.00%)	100.75 (+0.37%)	90 (0.00%)
**PBE+*U***	12.576 (+6.32%)	7.860 (+2.78%)	5.993 (+3.66%)	90 (0.00%)	93.27 (-7.09%)	90 (0.00%)
**PBE+D3-Grimme**	12.213 (+3.25%)	7.755 (+1.40%)	5.921 (2.43%)	90 (0.00%)	101.63 (+1.24%)	90 (0.00%)
**PBE+D3-BJ**	11.980 (+1.27%)	7.667 (+0.25%)	5.848 (+1.16%)	90 (0.00%)	101.41 (+1.24%)	90 (0.00%)
**PBE+*U*+D3-Grimme**	12.186 (+3.02)	7.891 (+3.18)	5.872 (+1.58)	90 (0.00%)	100.43 (+0.04%)	90 (0.00%)
**PBE+*U*+D3-BJ**	11.957 (+1.08%)	7.782 (+1.75%)	5.813 (+0.56%)	90 (0.00%)	100.15 (-0.24%)	90 (0.00%)
**PBEsol *U*+D3-Grimme**	11.853 (+0.21%)	7.630 (-0.23%)	5.737 (-0.77%)	90 (0.00%)	100.84 (+0.45%)	90 (0.00%)
**PBEsol *U*+D3-BJ**	11.584 (-2.07%)	7.516 (-1.73%)	5.691 (-1.55%)	90 (0.00%)	100.93 (+0.54%)	90 (0.00%)

Table S9: Comparison between the calculated and experimental bond and interaction distances of $Cs_2[UO_2Cl_4]$ system. All distances are reported in Angstroms (Å), while angles reported in degrees (°).

$Cs_2[UO_2Cl_4]{\cdot}2H_2O$	$U{=}O_{yl}$	U-Cl	$M{\cdots}Cl$	$M{\cdots}O_{yl}$
**Experimental[4]**	1.776	2.671	3.526	3.275
**PBE**	1.815	2.679	3.647	3.355
**PBE+*U***	1.772	2.724	3.624	4.098
**PBE+D3-Grimme**	1.817	2.667	3.637	3.215
**PBE+D3-BJ**	1.816	2.664	3.573	3.196
**PBE+*U*+D3-Grimme**	1.778	2.708	3.619	3.343
**PBE+*U*+D3-BJ**	1.778	2.704	3.559	3.332
**PBEsol+*U*+D3-Grimme**	1.771	2.671	3.486	3.218
**PBEsol+*U*+D3-BJ**	1.771	2.667	3.434	3.171

Table S10: Comparison between the calculated and experimental unit cell parameter, bond and interaction distances of $C_{10}H_{10}N_2[UO_2Cl_4]$ system. All distances are reported in Angstroms (Å), while angles reported in degrees (°).

$C_{10}H_{10}N_2[UO_2Cl_4]$	*a*	*b*	*c*	*α*	*β*	*γ*
**Experimental[5]**	5.567	8.572	8.839	71.14	73.08	86.53
**PBE+*U*+D3-Grimme**	5.478 (-1.59%)	8.159 (-0.62%)	8,884 (+0.51)	70.46 (-0.96%)	72.41 (-0.91%)	86.21 (-0.37%)
**PBE+*U*+D3-BJ**	5.486 (-1.45%)	8.583 (+0.13%)	8.889 (+0.56%)	70.84 (-0.42%)	72.32 (-1.04%)	86.20 (-0.39%)
**PBEsol+*U*+D3-Grimme**	5.344 (-4.01%)	8.422 (-1.75%)	8.784 (-0.62%)	70.38 (-1.07%)	71.52 (-2.13%)	86.43 (-0.11%)
**PBEsol+*U*+D3-BJ**	5.361 (-3.70%)	8.422 (-1.76%)	8.764 (-0.85%)	70.17 (-1.53%)	71.60 (-2.02%)	86.18 (-0.41%0

	$U{=}O_{yl}$	U-Cl	Cl···H
**Experimental[5]**	1.734	2.658, 2.668	2.569, 2.979
**PBE+*U*+D3-Grimme**	1.773	2.705, 2.710	2.379, 2.964
**PBE+*U*+D3-BJ**	1.773	2.703, 2.708	2.354, 2.967
**PBEsol+*U*+D3-Grimme**	1.765	2.672, 2.681	2.335, 2.831
**PBEsol+*U*+D3-BJ**	1.765	2.673, 2.678	2.306, 2.886

Table S11: Comparison between the calculated and experimental unit cell parameter, bond and interaction distances of $C_{12}H_{12}N_2[UO_2Cl_4]$ system. All distances are reported in Angstroms (Å), while angles reported in degrees (°).

$C_{12}H_{12}N_2[UO_2Cl_4]$	*a*	*b*	*c*	*α*	*β*	*γ*
**Experimental[5]**	7.000	8.176	8.444	81.59	73.86	66.80
**PBE+*U*+D3-Grimme**	6.996 (-0.06%)	8.157 (-0.23%)	8.375 (-0.81%)	80.90 (-0.84%)	73.91 (+0.07%)	66.00 (-1.20%)

**PBE+*U*+D3-BJ**	6.967 (-0.48%)	8.155 (-0.26%)	8.317 (-1.50%)	80.73 (-1.06%)	74.11 (+0.34%)	65.98 (-1.23%)
**PBEsol+*U*+D3-Grimme**	6.829 (-2.32%)	8.061 (-1.41%)	8.112 (-3.92%)	80.95 (-0.78%)	74.81 (+1.29%)	65.76 (-1.55%)
**PBEsol+*U*+D3-BJ**	6.829 (-2.44%)	8.061 (-1.41%)	8.112 (-3.92%)	80.95 (-0.78%)	74.813 (-1.29%)	65.766 (-1.54%)
	**$U=O_{yl}$**		**U-Cl**		**Cl···H**	
**Experimental[5]**	1.763		2.668, 2.676		2.642, 2.645	
**PBE+*U*+D3-Grimme**	1.774		2.697, 2.715		2.488, 2.549	
**PBE+*U*+D3-BJ**	1.774		2.695, 2.712		2.473, 2.530	
**PBEsol+*U*+D3-Grimme**	1.765		2.683, 2.665		2.401, 2.488	
**PBEsol+*U*+D3-BJ**	1.764		2.666, 2.683		2.406, 2.463	

Table S12: Comparison between the calculated and experimental unit cell parameter, bond and interaction distances of $C_{12}H_{14}N_2[UO_2Cl_4]$ system. All distances are reported in Angstroms (Å), while angles reported in degrees (°).

**$C_{12}H_{14}N_2[UO_2Cl_4]$**	***a***	***b***	***c***	***α***	***β***	***γ***
**Experimental[5]**	7.030	8.244	8.768	82.81	70.63	66.07
**PBE+*U*+D3-Grimme**	7.059 (+0.43%)	8.292 (+0.58%)	8.537 (-2.63%)	81.02 (+2.17%)	70.37 (0.37%)	65.29 (+1.18%)
**PBE+*U*+D3-BJ**	7.058 (+0.40%)	8.263 (0.24%)	8.600 (-1.91%)	81.85 (-1.15%)	70.54 (-0.12%)	65.71 (-0.55%)
**PBEsol+*U*+D3-Grimme**	6.901 (-1.82%)	8.193 (-0.61%)	8.363 (-4.61%)	81.61 (-1.45%)	70.55 (-0.37%)	64.81 (-2.06%)
**PBEsol+*U*+D3-BJ**	6.959 (-1.00%)	8.185 (-0.71%)	8.359 (-4.65%)	81.06 (-2.10%)	70.55 (-0.11%)	64.81 (-1.91%)
	**$U=O_{yl}$**		**U-Cl**		**Cl···H**	
**Experimental[5]**	1.762		2.655, 2.694		2.496, 2.825	
**PBE+*U*+D3-Grimme**	1.775		2.679		2.735	
**PBE+*U*+D3-BJ**	1.775		2.677, 2.734		2.204, 2.930	
**PBEsol+*U*+D3-Grimme**	1.766		2.648, 2.702		2.166, 2.821	
**PBEsol+*U*+D3-BJ**	1.766		2.703, 2.649		2.154, 2.844	

S4. Electronic structure

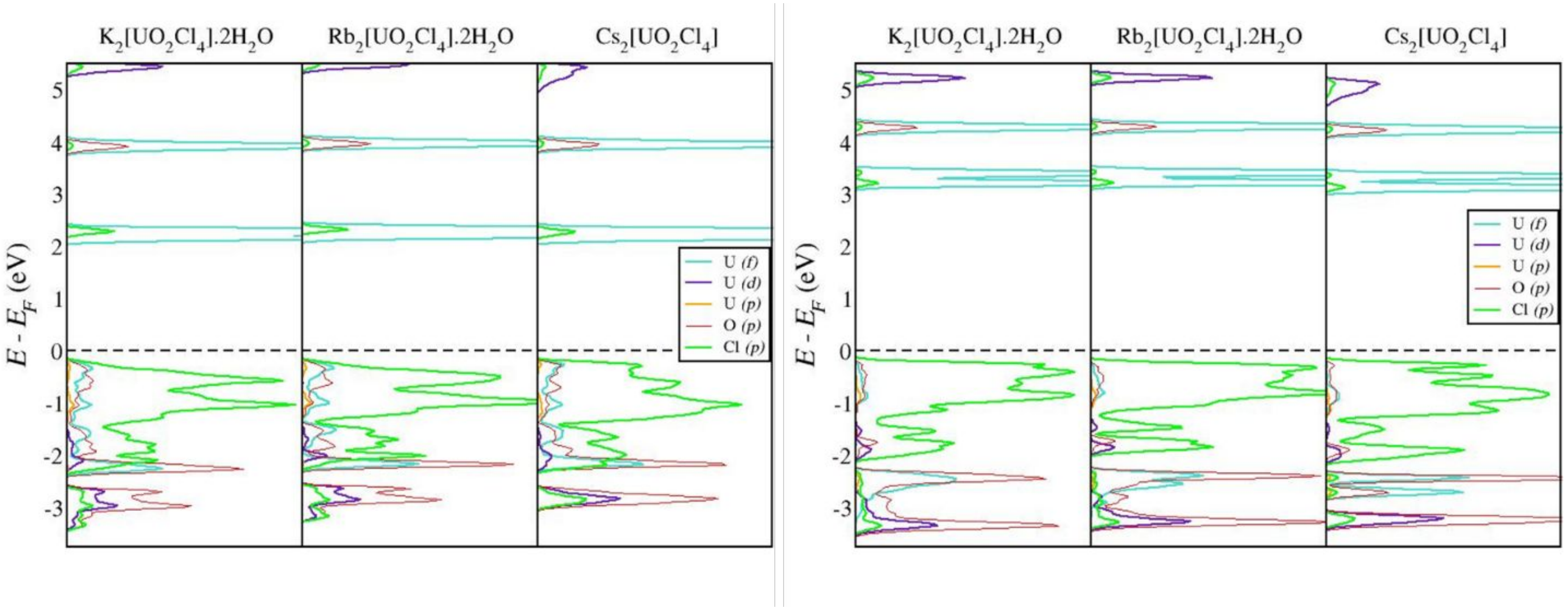

Figure S1: Calculated projected density of states for the uranyl-cation structures without (left) and with (right) a Hubbard *U* correction.

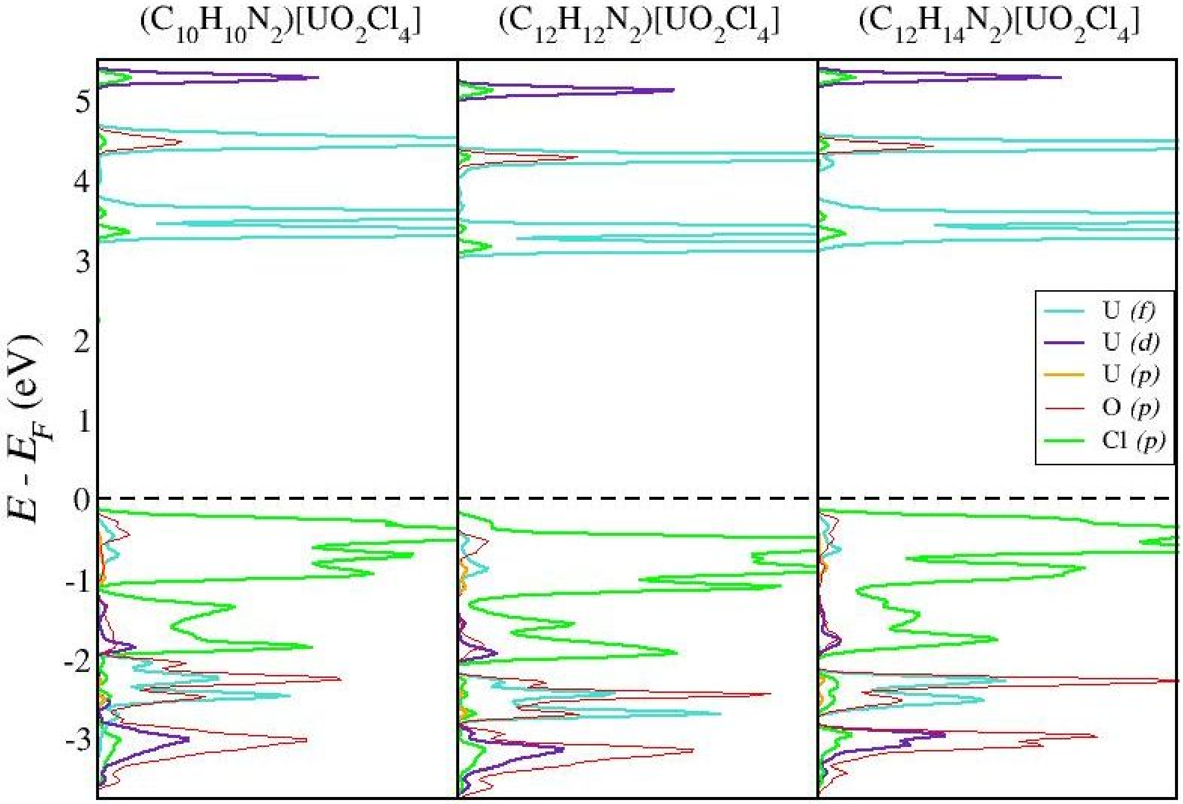

Figure S2. Calculated projected density of states for the uranyl-hydrogen structure

S5. Vibrational analysis

S5.1 Experimental data

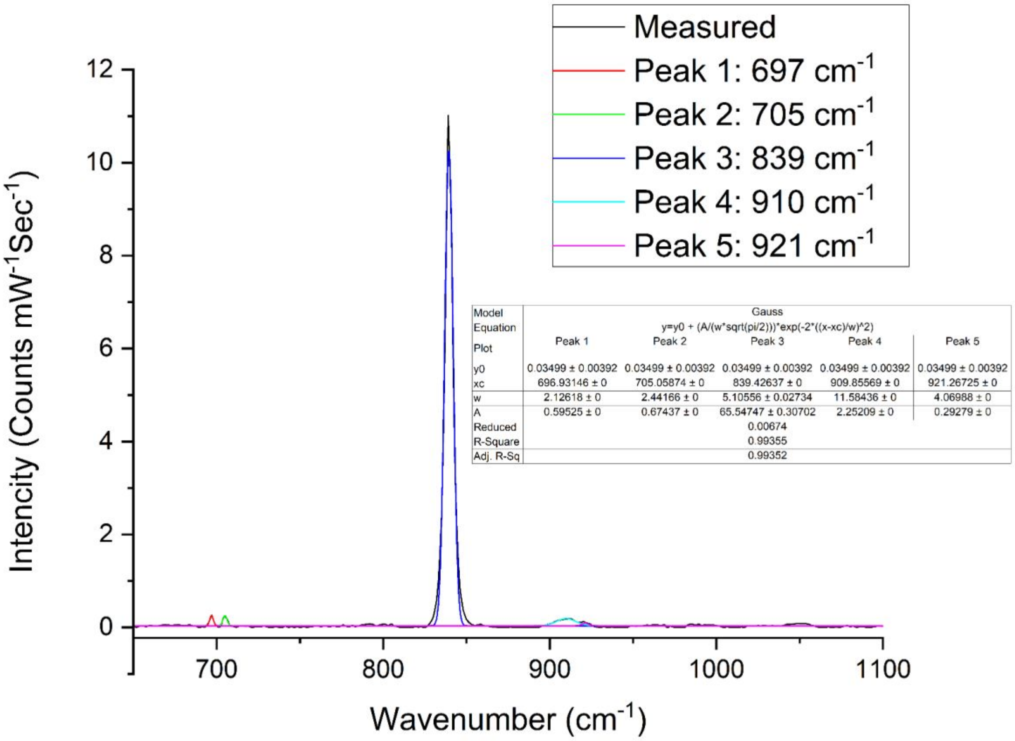

Figure S3: Fitted experimental Raman data of the powder $K_2[UO_2Cl_4]{\cdot}2H_2O$

S5.2 Calculated phonon frequencies, uranyl displacement amplitudes, and oxo displacement phase angle

Each crystal structure contained inversion symmetry. Therefore, vibrational modes resulting from atomic displacements which were asymmetric to the inversion center (ungerade; Mulliken symbol with subscript u) are IR active, while atomic displacements symmetric to the inversion center (gerade; Mulliken symbol with subscript g) are Raman active.

Table S13: Vibrational analysis of each phonon mode of $K_2[UO_2Cl_4]$. Modes corresponding to the symmetric (ν_1) and asymmetric (ν_3) stretch of the uranyl cation are highlighted in red.

		**PBE+*U*+D3-BJ**			**PBEsol+*U*+D3-BJ**		
**Mode**	**Mulliken Symbol**	**Energy (cm^{-1})**	s^n	θ_j^n	**Energy (cm^{-1})**	s^n	θ_j^n
0	A_u	-0.328	0.328	0	-0.283	0.327	0
1	A_u	-0.167	0.327	0	-0.230	0.326	0
2	A_u	0.024	0.326	0	-0.164	0.326	0
3	A_g	59.454	0.165	180	60.971	0.160	180
4	A_g	64.574	0.132	180	68.970	0.136	180
5	A_u	70.678	0.189	0	72.649	0.207	0
6	A_u	76.077	0.083	180	80.046	0.118	0
7	A_g	76.296	0.157	0	80.840	0.066	180
8	A_g	78.030	0.057	180	83.440	0.064	180
9	A_u	81.613	0.225	0	86.653	0.222	0
10	A_g	95.567	0.055	180	101.932	0.062	180
11	A_u	96.610	0.065	0	102.675	0.150	0
12	A_u	101.620	0.301	0	103.951	0.306	0
13	A_u	107.077	0.042	180	112.886	0.088	0
14	A_g	109.117	0.133	0	114.374	0.037	180
15	A_u	110.751	0.067	0	121.441	0.084	0
16	A_g	127.797	0.015	180	134.124	0.018	180
17	A_g	148.669	0.144	180	159.915	0.189	180
18	A_u	162.266	0.075	0	174.585	0.075	0
19	A_g	170.064	0.079	180	183.388	0.117	180
20	A_u	174.814	0.048	0	188.557	0.058	0
21	A_g	194.434	0.363	180	196.943	0.354	180
22	A_g	208.494	0.510	180	213.451	0.535	180
23	A_u	213.439	0.060	0	233.984	0.095	0
24	A_g	219.777	0.165	180	234.724	0.095	180
25	A_u	234.082	0.069	180	246.709	0.215	0
26	A_g	234.715	0.233	0	249.689	0.058	180
27	A_u	244.802	0.221	0	260.212	0.204	0
28	A_g	262.309	0.034	180	275.476	0.034	180
29	A_u	329.273	0.739	0	334.041	0.734	0
30	A_u	345.433	0.806	0	354.455	0.795	0
31	A_g	385.255	0.010	180	393.250	0.010	180
32	A_u	394.657	0.138	0	399.393	0.131	0
33	A_g	473.332	0.032	180	473.912	0.031	180
34	A_u	491.272	0.085	0	493.992	0.094	0
35	A_u	544.879	0.052	0	555.382	0.051	0
36	A_g	610.319	0.041	180	625.300	0.042	180
37	A_g	811.496	0.813	180	829.220	0.813	180
38	A_u	960.929	0.944	0	980.077	0.945	0
39	A_u	1589.401	0.007	0	1557.258	0.008	0
40	A_g	1593.476	0.001	180	1561.516	0.001	180
41	A_u	3515.619	0.001	0	3453.017	0.002	0
42	A_g	3518.119	0.002	180	3455.143	0.002	180

43	A_u	3605.915	0.001	0	3550.536	0.001	0
44	A_g	3606.592	0.001	180	3552.251	0.001	180

Table S14: Vibrational analysis of each phonon mode of $Rb_2[UO_2Cl_4].2H_2O$. Modes corresponding to the symmetric (ν_1) and asymmetric (ν_3) stretch of the uranyl cation are highlighted in red.

		**PBE+*U*+D3-BJ**			**PBEsol+*U*+D3-BJ**		
**Mode**	**Mulliken Symbol**	**Energy (cm^{-1})**	s^n	θ_j^n	**Energy (cm^{-1})**	s^n	θ_j^n
0	A_u	-0.535	0.303	0	-0.263	0.302	0
1	A_u	-0.182	0.301	0	-0.203	0.302	0
2	A_u	-0.130	0.302	0	-0.132	0.301	0
3	A_g	46.677	0.119	180	47.510	0.129	180
4	A_u	58.136	0.078	180	60.167	0.251	0
5	A_g	58.390	0.255	0	62.118	0.065	180
6	A_g	66.945	0.060	180	72.058	0.033	180
7	A_g	68.440	0.233	0	75.377	0.183	180
8	A_u	69.072	0.184	180	75.614	0.229	0
9	A_g	71.955	0.095	180	78.294	0.094	180
10	A_u	73.604	0.256	0	81.000	0.232	0
11	A_g	84.401	0.032	180	92.530	0.034	180
12	A_u	87.725	0.103	0	92.754	0.132	0
13	A_u	96.814	0.128	0	100.138	0.232	0
14	A_u	98.432	0.170	0	104.754	0.028	0
15	A_u	104.126	0.242	0	107.341	0.207	0
16	A_g	119.395	0.013	180	123.248	0.013	180
17	A_g	136.526	0.117	180	149.613	0.144	180
18	A_u	140.693	0.064	0	152.593	0.057	0
19	A_g	148.831	0.097	180	162.497	0.126	180
20	A_u	156.866	0.085	0	170.821	0.091	0
21	A_g	185.657	0.308	180	189.810	0.366	180
22	A_g	197.502	0.045	0	214.167	0.439	180
23	A_u	205.644	0.313	180	218.767	0.061	0
24	A_g	213.026	0.406	180	224.570	0.206	180
25	A_g	228.154	0.062	180	242.299	0.054	180
26	A_u	232.251	0.276	0	243.895	0.274	0
27	A_u	239.595	0.253	0	254.009	0.243	0
28	A_g	257.396	0.036	180	269.270	0.035	180
29	A_u	328.608	0.743	0	333.335	0.740	0
30	A_u	347.102	0.823	0	356.270	0.806	0
31	A_g	392.173	0.009	180	402.992	0.010	180
32	A_u	395.534	0.136	0	403.748	0.126	0
33	A_g	445.989	0.026	180	447.046	0.026	180
34	A_u	465.132	0.088	0	466.976	0.100	0
35	A_u	526.670	0.050	0	541.679	0.047	0
36	A_g	594.620	0.036	180	612.441	0.034	180

37	A_g	813.019	0.828	180	829.057	0.836	180
38	A_u	964.908	0.952	0	984.238	0.950	0
39	A_u	1594.054	0.007	0	1562.911	0.008	0
40	A_g	1595.636	0.001	180	1564.410	0.001	180
41	A_u	3518.449	0.001	0	3457.725	0.002	0
42	A_g	3520.997	0.002	180	3461.060	0.002	180
43	A_u	3616.516	0.001	0	3560.781	0.001	0
44	A_g	3617.464	0.001	180	3562.609	0.001	180

Table S15: Vibrational analysis of each phonon mode of $Cs_2[UO_2Cl_4]$. Modes corresponding to the symmetric (ν_1) and asymmetric (ν_3) stretch of the uranyl cation are highlighted in red.

		PBE+*U*+D3-BJ					PBEsol+*U*+D3-BJ				
Mode	Mulliken Symbol	Energy (cm^{-1})	s^n	θ_j^n	s^n	θ_j^n	Energy (cm^{-1})	s^n	θ_j^n	s^n	θ_j^n
0	A_u	-1.178	0.164	0	0.164	0	-2.877	0.160	0	0.160	0
1	A_u	-0.264	0.166	0	0.166	0	-0.689	0.166	0	0.166	0
2	B_u	-0.210	0.166	0	0.166	0	-0.269	0.167	0	0.167	0
3	B_u	25.257	0.169	0	0.169	0	29.887	0.208	0	0.208	0
4	B_u	29.556	0.215	0	0.215	0	32.032	0.178	0	0.178	0
5	B_u	33.751	0.046	180	0.046	180	32.494	0.054	180	0.054	180
6	B_g	41.433	0.067	180	0.067	180	37.204	0.071	180	0.071	180
7	B_g	43.928	0.042	180	0.042	180	48.060	0.059	180	0.059	180
8	A_g	44.965	0.051	180	0.051	180	50.640	0.224	0	0.224	0
9	A_g	45.648	0.200	0	0.200	0	50.690	0.048	180	0.048	180
10	A_u	50.248	0.021	180	0.021	180	55.054	0.179	0	0.179	0
11	A_u	52.463	0.149	0	0.149	0	57.414	0.007	180	0.007	180
12	A_g	53.157	0.103	180	0.103	180	60.660	0.123	0	0.123	0
13	A_g	53.305	0.167	0	0.167	0	61.741	0.105	180	0.105	180
14	B_u	56.166	0.206	0	0.206	0	62.215	0.118	0	0.118	0
15	B_u	56.899	0.179	0	0.179	0	62.305	0.215	0	0.215	0
16	B_u	59.086	0.093	0	0.093	0	63.983	0.179	0	0.179	0
17	B_u	59.331	0.176	0	0.176	0	69.595	0.196	0	0.196	0
18	B_g	64.476	0.024	180	0.024	180	72.610	0.018	180	0.018	180
19	B_g	67.527	0.062	180	0.062	180	77.953	0.080	180	0.080	180
20	B_g	73.739	0.002	180	0.002	180	86.171	0.004	180	0.004	180
21	B_g	75.942	0.084	180	0.084	180	87.094	0.077	180	0.077	180
22	A_g	79.773	0.109	180	0.109	180	88.439	0.113	180	0.113	180
23	A_g	88.922	0.114	180	0.114	180	98.617	0.119	180	0.119	180
24	A_u	98.287	0.064	0	0.064	0	108.479	0.050	0	0.050	0
25	A_u	99.272	0.053	0	0.053	0	110.248	0.065	0	0.065	0
26	B_u	103.222	0.078	0	0.078	0	110.437	0.037	0	0.037	0

27	B_u	103.907	0.097	0	0.097	0	110.449	0.089	0	0.089	0
28	B_u	108.911	0.040	0	0.040	0	116.391	0.123	0	0.123	0
29	B_u	111.718	0.141	0	0.141	0	116.570	0.040	0	0.040	0
30	A_u	120.608	0.006	0	0.006	0	128.995	0.010	0	0.010	0
31	A_u	121.045	0.004	180	0.004	180	129.582	0.006	180	0.006	180
32	A_g	123.697	0.109	0	0.109	0	129.941	0.095	0	0.095	0
33	A_g	129.082	0.006	180	0.006	180	136.477	0.003	180	0.003	180
34	B_g	191.663	0.291	180	0.291	180	189.942	0.287	180	0.287	180
35	B_g	193.997	0.285	180	0.285	180	192.163	0.276	180	0.276	180
36	A_g	197.228	0.283	180	0.283	180	197.023	0.275	180	0.275	180
37	A_g	197.453	0.278	180	0.278	180	197.721	0.265	180	0.265	180
38	B_g	218.877	0.042	180	0.042	180	234.368	0.025	180	0.025	180
39	B_g	220.117	0.013	180	0.013	180	235.605	0.010	180	0.010	180
40	A_u	232.348	0.136	0	0.136	0	247.446	0.139	0	0.139	0
41	A_u	233.429	0.138	0	0.138	0	247.675	0.144	0	0.144	0
42	B_u	236.757	0.128	0	0.128	0	253.507	0.131	0	0.131	0
43	B_u	244.191	0.137	0	0.137	0	259.218	0.143	0	0.143	0
44	A_g	253.471	0.021	180	0.021	180	267.502	0.021	180	0.021	180
45	A_g	259.401	0.025	180	0.025	180	272.722	0.024	180	0.024	180
46	B_u	329.475	0.485	0	0.485	0	333.449	0.469	0	0.469	0
47	B_u	333.418	0.438	0	0.438	0	337.989	0.418	0	0.418	0
48	A_u	335.203	0.471	0	0.471	0	338.719	0.454	0	0.454	0
49	A_u	338.154	0.449	0	0.449	0	342.045	0.431	0	0.431	0
50	A_g	804.852	0.459	180	0.459	180	813.291	0.458	180	0.458	180
51	A_g	805.850	0.463	180	0.463	180	819.696	0.461	180	0.461	180
52	B_u	973.137	0.494	0	0.494	0	986.226	0.494	0	0.494	0
53	B_u	979.789	0.486	0	0.486	0	993.506	0.486	0	0.486	0

Table S16: Vibrational analysis of each phonon mode of $C_{10}H_{10}N_2[UO_2Cl_4]$. Modes corresponding to the symmetric (ν_1) and asymmetric (ν_3) stretch of the uranyl cation, along with combination modes of the N-heterocyclic cation are highlighted in red.

		PBE+*U*+D3-BJ			PBEsol+*U*+D3-BJ		
**Mode**	**Mulliken Symbol**	**Energy (cm^{-1})**	s^n	θ_j^n	**Energy (cm^{-1})**	s^n	θ_j^n
0	A_u	-0.404	0.237	0	-0.324	0.234	0
1	A_u	-0.219	0.236	0	-0.160	0.235	0
2	A_u	-0.178	0.236	0	-0.092	0.235	0
3	A_u	50.946	0.087	0	58.171	0.156	0
4	A_g	54.887	0.057	180	58.516	0.056	180
5	A_u	57.382	0.183	0	59.666	0.134	0
6	A_g	64.747	0.128	180	70.292	0.142	180
7	A_u	70.133	0.163	0	73.165	0.168	0
8	A_g	80.525	0.069	180	86.834	0.057	180
9	A_u	85.758	0.122	0	90.506	0.122	0
10	A_g	87.025	0.047	180	92.902	0.036	180
11	A_u	97.462	0.139	0	103.392	0.127	0
12	A_g	103.209	0.098	180	112.910	0.092	180
13	A_u	110.273	0.107	0	114.957	0.109	0
14	A_g	116.201	0.039	180	123.245	0.040	180
15	A_u	124.127	0.053	0	128.053	0.051	0
16	A_g	126.221	0.064	180	131.221	0.073	180
17	A_u	130.611	0.043	0	137.896	0.041	0
18	A_u	141.966	0.089	0	151.050	0.077	0
19	A_g	185.569	0.023	0	186.498	0.482	180
20	A_u	185.855	0.495	180	188.205	0.026	0
21	A_g	220.867	0.034	180	224.478	0.030	180
22	A_g	224.472	0.544	180	230.350	0.546	180
23	A_u	230.032	0.245	0	240.974	0.242	0
24	A_g	235.943	0.234	0	244.837	0.027	180
25	A_u	236.300	0.021	180	247.065	0.238	0
26	A_g	259.369	0.038	180	268.280	0.036	180
27	A_g	333.798	0.022	180	336.648	0.027	180
28	A_u	334.028	0.843	0	339.480	0.810	0
29	A_u	341.885	0.869	0	348.426	0.845	0
30	A_u	379.969	0.008	0	377.476	0.009	0
31	A_g	391.514	0.002	180	388.909	0.002	180
32	A_g	420.161	0.006	180	420.566	0.007	180
33	A_u	430.484	0.017	0	428.436	0.020	0
34	A_g	557.447	0.002	180	557.579	0.002	180
35	A_u	612.559	0.003	0	609.057	0.003	0
36	A_g	630.799	0.003	180	627.050	0.004	180
37	A_u	649.493	0.004	0	645.655	0.005	0

38	A_u	708.071	0.002	0	709.093	0.002	0
39	A_g	717.125	0.012	180	716.057	0.012	180
40	A_g	762.625	0.005	180	764.630	0.005	180
41	A_u	771.617	0.023	0	768.202	0.023	0
42	A_g	807.681	0.240	180	809.129	0.145	180
43	A_g	824.586	0.293	180	833.495	0.414	180
44	A_u	856.013	0.016	0	851.506	0.014	0
45	A_g	879.881	0.008	180	876.318	0.011	180
46	A_u	915.592	0.047	0	913.331	0.043	0
47	A_g	922.164	0.018	180	918.766	0.025	180
48	A_u	976.699	0.543	0	980.657	0.092	0
49	A_u	987.124	0.092	0	990.779	0.505	0
50	A_g	996.567	0.003	180	991.531	0.003	180
51	A_g	998.715	0.012	0	995.178	0.003	180
52	A_u	999.444	0.002	180	996.497	0.003	0
53	A_g	1011.782	0.007	180	1009.869	0.009	180
54	A_u	1013.010	0.037	0	1010.827	0.082	0
55	A_u	1036.727	0.020	0	1038.247	0.029	0
56	A_u	1057.108	0.019	0	1054.492	0.025	0
57	A_g	1072.321	0.004	180	1073.537	0.005	180
58	A_g	1100.918	0.003	180	1091.081	0.003	180
59	A_u	1119.042	0.011	0	1116.154	0.015	0
60	A_u	1192.299	0.021	0	1175.500	0.029	0
61	A_g	1219.713	0.002	180	1201.292	0.013	180
62	A_g	1228.260	0.018	180	1209.218	0.021	180
63	A_u	1233.661	0.032	0	1213.498	0.048	0
64	A_g	1280.886	0.000	180	1288.065	0.001	180
65	A_u	1303.414	0.005	0	1297.993	0.003	0
66	A_g	1324.154	0.000	180	1308.851	0.002	180
67	A_g	1352.872	0.004	180	1371.081	0.004	180
68	A_u	1378.553	0.005	0	1387.488	0.006	0
69	A_u	1458.750	0.004	0	1450.587	0.005	0
70	A_u	1481.832	0.002	0	1472.155	0.003	0
71	A_g	1502.049	0.003	180	1491.374	0.004	180
72	A_g	1519.181	0.001	180	1515.950	0.001	180
73	A_u	1579.670	0.006	0	1582.342	0.007	0
74	A_g	1586.649	0.004	180	1593.673	0.004	180
75	A_u	1628.200	0.001	0	1634.919	0.001	0
76	A_g	1631.210	0.001	180	1638.800	0.001	180
77	A_u	3090.833	0.000	0	3045.210	0.000	0
78	A_g	3092.776	0.000	180	3046.918	0.000	180
79	A_g	3100.838	0.000	180	3057.353	0.000	180
80	A_u	3102.258	0.001	0	3058.688	0.001	0
81	A_u	3109.798	0.001	0	3065.012	0.001	0
82	A_g	3111.633	0.000	180	3067.534	0.001	180

83	A_u	3139.196	0.001	0	3096.544	0.001	0
84	A_g	3140.148	0.001	180	3097.389	0.001	180
85	A_u	3246.448	0.003	0	3187.186	0.003	0
86	A_g	3249.775	0.001	180	3190.747	0.001	180

Table S17: Vibrational analysis of each phonon mode of $C_{12}H_{12}N_2[UO_2Cl_4]$. Modes corresponding to the symmetric (ν_1) and asymmetric (ν_3) stretch of the uranyl cation, along with combination modes of the N-heterocyclic cation are highlighted in red.

		**PBE+*U*+D3-BJ**			**PBEsol+*U*+D3-BJ**		
**Mode**	**Mulliken Symbol**	**Energy (cm^{-1})**	s^n	θ_j^n	**Energy (cm^{-1})**	s^n	θ_j^n
0	A_u	-0.376	0.216	0	-0.326	0.217	0
1	---	-0.237	0.216	0	-0.158	0.216	0
2	---	-0.116	0.216	0	-0.144	0.216	0
3	A_g	41.130	0.141	180	42.072	0.140	180
4	A_g	65.123	0.136	180	67.583	0.131	180
5	A_u	68.309	0.140	0	72.015	0.136	0
6	A_u	72.375	0.048	180	76.956	0.176	0
7	A_g	73.475	0.175	0	79.976	0.056	180
8	A_u	79.203	0.126	0	83.050	0.127	0
9	A_g	82.761	0.013	180	88.011	0.006	180
10	A_u	90.352	0.065	0	94.749	0.099	0
11	A_g	95.359	0.057	180	102.267	0.048	180
12	A_u	99.862	0.140	0	104.343	0.142	0
13	A_u	102.482	0.060	0	106.654	0.045	0
14	A_g	105.973	0.012	180	112.414	0.016	180
15	A_u	110.806	0.091	0	114.706	0.103	0
16	A_u	113.725	0.040	0	119.147	0.041	0
17	A_g	121.112	0.106	0	126.965	0.028	180
18	A_u	123.475	0.019	180	127.040	0.080	0
19	A_g	129.867	0.003	180	136.566	0.004	180
20	A_u	131.870	0.016	0	140.705	0.017	0
21	A_g	194.715	0.510	180	194.173	0.510	180
22	A_g	207.689	0.490	180	209.385	0.497	180
23	A_g	211.649	0.025	180	214.566	0.016	180
24	A_g	227.292	0.045	180	237.923	0.040	180
25	A_u	230.400	0.211	0	239.870	0.191	0
26	A_g	235.193	0.016	180	240.657	0.015	180
27	A_u	237.564	0.230	0	248.493	0.221	0
28	A_g	257.664	0.027	180	266.583	0.028	180
29	A_u	273.598	0.038	0	274.937	0.051	0
30	A_g	292.820	0.006	180	294.254	0.008	180
31	A_u	336.828	0.849	0	342.660	0.805	0
32	A_u	346.406	0.807	0	352.674	0.770	0

33	A_u	396.265	0.023	0	392.932	0.031	0
34	A_g	397.964	0.001	180	395.149	0.001	180
35	A_g	467.701	0.005	180	465.614	0.006	180
36	A_u	469.516	0.003	0	466.547	0.004	0
37	A_u	524.550	0.004	0	522.447	0.004	0
38	A_u	538.187	0.002	0	535.883	0.002	0
39	A_g	634.368	0.001	180	630.359	0.001	180
40	A_u	639.593	0.001	0	635.194	0.001	0
41	A_g	642.489	0.002	180	637.980	0.001	180
42	A_g	698.575	0.008	180	697.572	0.009	180
43	A_u	719.141	0.004	0	714.603	0.005	0
44	A_g	739.489	0.016	180	730.875	0.015	180
45	A_u	757.711	0.008	0	753.449	0.008	0
46	A_g	820.701	0.580	180	828.786	0.099	180
47	A_u	838.861	0.003	0	831.270	0.006	0
48	A_g	838.953	0.048	180	838.209	0.420	180
49	A_g	842.388	0.006	0	844.005	0.022	180
50	A_u	850.840	0.003	180	844.421	0.005	0
51	A_u	878.392	0.023	180	874.362	0.009	0
52	A_g	881.709	0.009	0	877.555	0.034	180
53	A_g	903.561	0.004	180	897.023	0.002	180
54	A_u	947.325	0.024	0	932.080	0.016	0
55	A_u	966.542	0.000	180	956.786	0.002	0
56	A_g	966.590	0.004	0	957.065	0.001	180
57	A_g	970.225	0.003	180	961.220	0.004	180
58	A_u	974.949	0.020	0	965.308	0.009	0
59	A_u	986.606	0.618	0	1001.002	0.248	0
60	A_g	1003.707	0.006	180	1003.539	0.006	180
61	A_u	1004.980	0.080	0	1007.851	0.362	0
62	A_u	1056.828	0.014	0	1054.330	0.016	0
63	A_g	1057.051	0.005	180	1055.940	0.006	180
64	A_u	1088.492	0.021	0	1078.988	0.038	0
65	A_g	1092.672	0.007	180	1082.676	0.009	180
66	A_u	1183.669	0.028	0	1166.650	0.040	0
67	A_g	1185.410	0.006	180	1166.685	0.009	180
68	A_g	1206.217	0.000	180	1207.063	0.000	180
69	A_u	1233.669	0.006	0	1219.330	0.007	0
70	A_g	1237.999	0.003	180	1221.880	0.004	180
71	A_u	1241.141	0.012	0	1225.965	0.016	0
72	A_u	1280.783	0.002	0	1280.151	0.001	0
73	A_g	1301.562	0.001	180	1287.787	0.001	180
74	A_g	1326.634	0.001	180	1308.090	0.001	180
75	A_u	1337.745	0.002	0	1321.148	0.003	0
76	A_u	1370.129	0.002	180	1387.561	0.003	0
77	A_g	1370.651	0.004	0	1387.609	0.002	180

78	A_g	1475.206	0.001	180	1468.302	0.002	180
79	A_u	1487.653	0.001	180	1480.967	0.004	0
80	A_g	1487.752	0.003	0	1483.084	0.002	180
81	A_u	1492.927	0.002	0	1488.230	0.002	0
82	A_g	1577.048	0.001	180	1580.933	0.002	180
83	A_u	1577.836	0.002	0	1581.723	0.002	0
84	A_g	1614.433	0.001	180	1622.398	0.001	180
85	A_u	1619.094	0.002	0	1626.088	0.003	0
86	A_g	1640.027	0.001	180	1645.828	0.001	180
87	A_g	3102.528	0.000	180	3066.606	0.000	180
88	A_u	3112.919	0.000	0	3077.298	0.000	0
89	A_g	3135.221	0.000	0	3098.975	0.000	180
90	A_u	3135.554	0.000	180	3099.058	0.000	0
91	A_g	3136.480	0.000	180	3101.423	0.000	180
92	A_u	3136.977	0.000	0	3101.668	0.000	0
93	A_u	3159.919	0.001	0	3122.175	0.000	0
94	A_g	3160.231	0.000	180	3122.702	0.000	180
95	A_u	3161.230	0.000	0	3126.170	0.001	0
96	A_g	3162.191	0.000	180	3126.928	0.000	180
97	A_u	3278.705	0.001	0	3217.658	0.002	0
98	A_g	3282.066	0.002	180	3220.796	0.002	180

Table S18: Vibrational analysis of each phonon mode of $C_{12}H_{14}N_2[UO_2Cl_4]$. Modes corresponding to the symmetric (ν_1) and asymmetric (ν_3) stretch of the uranyl cation, along with combination modes of the N-heterocyclic cation are highlighted in red.

		**PBE+*U*+D3-BJ**			**PBEsol+*U*+D3-BJ**		
**Mode**	**Mulliken Symbol**	**Energy (cm^{-1})**	s^n	θ_j^n	**Energy (cm^{-1})**	s^n	θ_j^n
0	Au	-0.53	0.21	0	-0.44	0.21	0
1	A_u	-0.21	0.21	0	-0.34	0.21	0
2	A_u	-0.17	0.21	0	-0.18	0.21	0
3	A_g	48.66	0.16	180	51.24	0.15	180
4	A_u	55.64	0.15	0	58.00	0.15	0
5	A_g	56.62	0.10	180	59.39	0.09	180
6	A_u	60.67	0.13	0	63.53	0.13	0
7	A_g	67.63	0.03	180	69.03	0.03	180
8	A_u	75.61	0.12	0	78.89	0.11	0
9	A_u	77.85	0.10	0	81.98	0.10	0
10	A_g	83.17	0.07	180	88.52	0.08	180
11	A_u	89.40	0.13	0	91.40	0.14	0
12	A_g	90.03	0.01	180	96.56	0.02	180
13	A_u	95.13	0.10	0	98.73	0.08	0
14	A_g	98.57	0.02	180	103.21	0.03	180
15	A_g	102.45	0.02	180	106.97	0.03	180

16	A_u	113.39	0.06	0	116.43	0.10	0
17	A_u	116.19	0.12	0	121.01	0.10	0
18	A_g	131.20	0.02	180	136.47	0.02	180
19	A_u	138.50	0.04	0	146.99	0.03	0
20	A_u	140.54	0.04	0	149.77	0.03	0
21	A_g	153.51	0.02	180	159.24	0.02	180
22	A_g	195.37	0.53	180	194.97	0.52	180
23	A_g	207.31	0.50	180	208.07	0.48	180
24	A_g	219.04	0.07	180	228.50	0.05	180
25	A_u	220.41	0.27	0	230.36	0.26	0
26	A_g	238.96	0.01	180	240.84	0.01	180
27	A_u	242.77	0.28	0	252.82	0.25	0
28	A_g	261.31	0.03	180	270.56	0.03	180
29	A_u	277.15	0.03	0	275.35	0.04	0
30	A_g	319.02	0.00	180	318.01	0.00	180
31	A_u	336.14	0.85	0	341.78	0.81	0
32	A_u	344.96	0.79	0	350.81	0.75	0
33	A_u	368.62	0.01	0	367.23	0.01	0
34	A_u	396.53	0.02	0	394.16	0.03	0
35	A_g	396.76	0.00	180	394.57	0.00	180
36	A_g	476.03	0.00	180	475.19	0.01	180
37	A_u	518.86	0.00	0	517.14	0.00	0
38	A_u	529.45	0.00	0	527.95	0.00	0
39	A_g	606.30	0.01	180	603.77	0.01	180
40	A_g	641.93	0.00	180	638.16	0.00	180
41	A_u	643.32	0.00	0	639.51	0.00	0
42	A_g	723.69	0.01	180	724.57	0.01	180
43	A_u	729.57	0.00	0	728.43	0.00	0
44	A_g	768.86	0.04	180	763.31	0.03	180
45	A_u	779.49	0.00	0	771.84	0.00	0
46	A_u	779.97	0.00	0	774.47	0.00	0
47	A_u	811.80	0.00	0	815.70	0.00	0
48	A_g	817.77	0.63	180	832.56	0.45	180
49	A_u	848.00	0.00	0	838.75	0.01	0
50	A_g	849.78	0.03	180	841.96	0.11	180
51	A_g	863.81	0.00	180	863.64	0.00	180
52	A_g	909.96	0.00	180	907.34	0.02	0
53	A_u	912.09	0.02	0	908.32	0.00	180
54	A_g	967.95	0.00	180	958.31	0.00	180
55	A_u	971.43	0.02	0	962.15	0.01	0
56	A_g	979.07	0.00	180	969.90	0.00	180
57	A_u	980.82	0.28	0	975.12	0.04	0
58	A_g	980.99	0.00	180	975.68	0.00	180
59	A_u	984.52	0.27	0	999.40	0.00	180
60	A_g	987.64	0.00	180	999.68	0.49	0

61	A_g	1008.77	0.01	180	1008.61	0.01	180
62	A_u	1009.17	0.06	0	1009.78	0.14	0
63	A_u	1061.13	0.03	0	1058.26	0.04	0
64	A_g	1061.37	0.00	180	1059.27	0.00	180
65	A_u	1079.32	0.02	0	1072.10	0.03	0
66	A_g	1097.85	0.01	180	1086.51	0.01	180
67	A_u	1148.70	0.01	0	1133.62	0.01	0
68	A_g	1186.22	0.01	180	1169.38	0.01	180
69	A_u	1186.92	0.03	0	1171.16	0.04	0
70	A_u	1213.11	0.00	0	1208.60	0.00	0
71	A_g	1215.41	0.00	180	1216.12	0.00	180
72	A_g	1254.97	0.00	180	1239.72	0.00	180
73	A_u	1257.40	0.01	0	1243.38	0.01	0
74	A_g	1263.77	0.00	180	1251.94	0.00	180
75	A_u	1265.56	0.00	0	1255.24	0.00	0
76	A_g	1316.81	0.00	180	1304.41	0.00	180
77	A_u	1324.54	0.00	0	1309.24	0.00	0
78	A_g	1331.65	0.00	180	1314.97	0.00	180
79	A_u	1371.26	0.00	0	1388.83	0.00	0
80	A_g	1372.29	0.00	180	1389.66	0.00	180
81	A_g	1438.92	0.00	180	1411.75	0.00	180
82	A_u	1453.31	0.00	0	1426.54	0.00	0
83	A_u	1488.62	0.00	0	1483.02	0.00	0
84	A_g	1490.45	0.00	180	1484.87	0.00	180
85	A_u	1502.60	0.00	0	1495.50	0.00	0
86	A_g	1504.19	0.00	180	1496.95	0.00	180
87	A_u	1591.82	0.00	0	1595.37	0.00	0
88	A_g	1592.80	0.00	180	1596.97	0.00	180
89	A_u	1625.66	0.00	0	1633.10	0.00	0
90	A_g	1631.14	0.00	180	1638.38	0.00	180
91	A_g	2966.08	0.00	180	2929.97	0.00	180
92	A_u	2979.61	0.00	0	2944.52	0.00	0
93	A_g	3017.57	0.00	180	2983.65	0.00	180
94	A_u	3037.29	0.00	0	3003.71	0.00	0
95	A_u	3120.43	0.00	0	3084.67	0.00	0
96	A_g	3120.47	0.00	180	3084.69	0.00	180
97	A_g	3126.87	0.00	180	3090.42	0.00	180
98	A_u	3127.04	0.00	0	3090.77	0.00	0
99	A_u	3157.23	0.00	0	3104.20	0.00	180
100	A_g	3157.43	0.00	180	3104.41	0.00	0
101	A_u	3163.77	0.00	0	3124.83	0.00	0
102	A_g	3164.30	0.00	180	3125.38	0.00	180
103	A_g	3170.71	0.00	180	3134.43	0.00	0
104	A_u	3170.76	0.00	0	3134.48	0.00	180

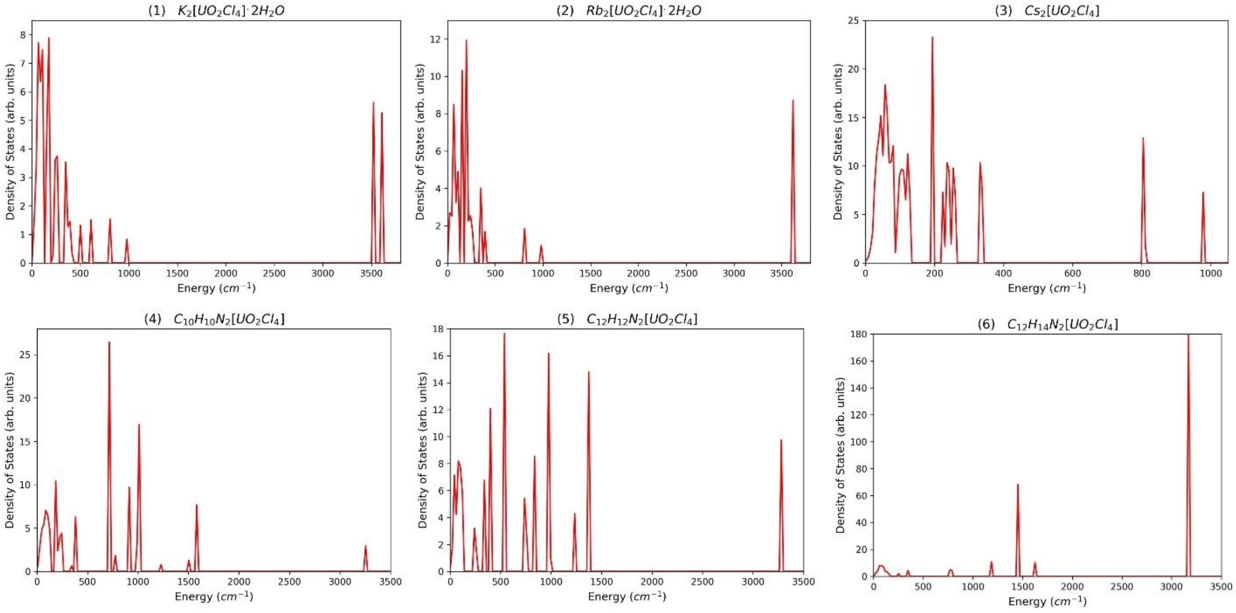

Figure S4. Calculated phonon densities of states for each of the uranyl-cation and uranyl-hydrogen structures.

S5.3 Eigenvector projections of the calculated phonon modes.

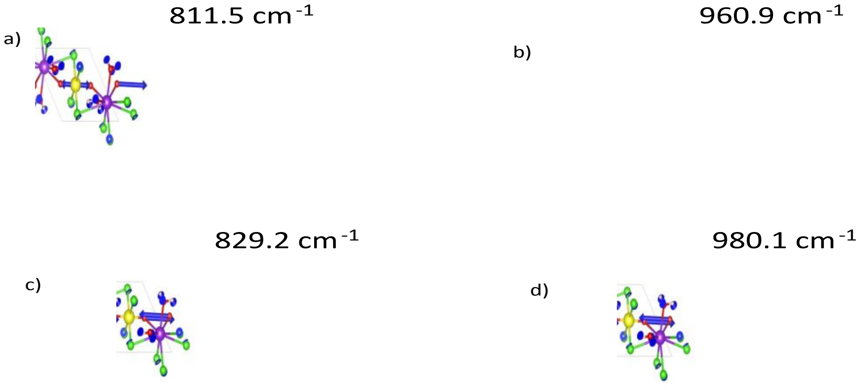

Figure S5: Phonon eigenvectors of the symmetric (ν_1) (left) and asymmetric (ν_3) (right) stretch of the uranyl cation in $K_2[UO_2Cl_4].2H_2O$. Figures A and B correspond PBE+*U*+BJ, while Figures C and D correspond to PBEsol+*U*+BJ.

a) 813.0 cm^{-1}

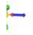

b) 964.9 cm^{-1}

c) 829.0 cm^{-1}

d) 984.2 cm^{-1}

Figure S6: Phonon eigenvectors of the symmetric (ν_1) (left) and asymmetric (ν_3) (right) stretch of the uranyl cation in $Rb_2[UO_2Cl_4].2H_2O$. Figures A and B correspond PBE+*U*+BJ, while Figures C and D correspond to PBEsol+*U*+BJ.

a) 804.9 cm^{-1}

b) 805.9cm $^{-1}$

c) 973.1 cm^{-1}

d) 879.8cm $^{-1}$

e) 813.3 cm^{-1}

f) 819.7cm $^{-1}$

g) 986.2 cm^{-1}

h) 993.5 cm^{-1}

Figure S7: Phonon eigenvectors of the symmetric (ν_1) (left) and asymmetric (ν_3) (right) stretch of the uranyl cation in $Cs_2[UO_2Cl_4]$. Figures A-D correspond PBE+*U*+BJ, while Figures E-H correspond to PBEsol+*U*+BJ.

807.7 cm^{-1}

a)

824.6cm $^{-1}$

b)

996.7cm $^{-1}$

c)

809.1 cm^{-1}

d)

833.5cm $^{-1}$

e)

990.8 cm^{-1}

f)

Figure S8: Phonon eigenvectors of the symmetric (ν_1), asymmetric (ν_3), and combination modes observed in $C_{10}H_{10}N_2[UO_2Cl_4]$. (Highlighted red in Table S14). Figures A-C correspond PBE+*U*+BJ, while Figures D-F correspond to PBEsol+*U*+BJ.

820.7 cm^{-1}

a)

986.6 cm^{-1}

b)

838.2 cm^{-1}

c)

1001.0 cm^{-1}

d)

1007.9 cm^{-1}

e)

Figure S9: Phonon eigenvectors of the symmetric (ν_1), asymmetric (ν_3), and combination modes observed in $C_{12}H_{12}N_2[UO_2Cl_4]$. (Highlighted red in Table S15). Figures A-B correspond PBE+*U*+BJ, while Figures C-E correspond to PBEsol+*U*+BJ.

817.8 cm^{-1}

980.8cm^{-1}

984.5 cm^{-1}

a)

b)

c)

832.6 cm^{-1}

842.0cm^{-1}

999.7cm^{-1}

d)

e)

f)

Figure S10: Phonon eigenvectors of the symmetric (ν_1), asymmetric (ν_3), and combination modes observed in $C_{12}H_{14}N_2[UO_2Cl_4]$. (Highlighted red in Table S14). Figures A-C correspond PBE+*U*+BJ, while Figures D-F correspond to PBEsol+*U*+BJ.

S6. Formation Enthalpies

S6.1 DFT + Thermodynamics

The thermocycles and energies for the formation enthalpy calculations in each uranyl hybrid structure are shown below. Standard state formation enthalpies for $HCl_{(aq)}$ and $H_2O_{(l)}$ were taken from Ref [6]. To calculate the energies for gaseous phase systems ($H_{2(g)}$, $O_{2(g)}$, and $Cl_{2(g)}$), each system was run in a large box (Table S3) with their calculated zero-point energies added to the DFT total energy. An additional correction was applied to the energy to account for the change from in temperature from 0 K → 298.15 K as obtained from the NIST-JANAF thermochemical tables. A correction was also applied to the $O_{2(g)}$ molecule due to its over-binding using GGA-PBE was done previously in literature.[7-10]

$K_2[UO_2Cl_4]{\cdot}2H_2O$

ΔH₁: $UO_{3(s)} + 2KCl_{(s)} + 2H_{2(g)} + Cl_{2(g)} + \frac{1}{2}O_{2(g)} \rightarrow K_2[UO_2Cl_4]\square 2H_2O$ **[-8.05 eV]**

ΔH₂: $\frac{1}{2}H_{2(g)} + \frac{1}{2}Cl_{2(g)} \rightarrow HCl_{(aq)}$ **[-1.73 eV]**

ΔH₃: $H_{2(g)} + \frac{1}{2}O_{2(g)} \rightarrow H_2O_{(l)}$ **[-2.96 eV]**

Overall: (ΔH₁ + -2ΔH₂ + -ΔH₃) $UO_{3(s)} + 2KCl_{(s)} + 2HCl_{(aq)} + H_2O_{(l)} \rightarrow K_2[UO_2Cl_4]\square 2H_2O$ **[-1.63 eV]**

$Rb_2[UO_2Cl_4]{\cdot}2H_2O$

ΔH₁: $UO_{3(s)} + 2RbCl_{(s)} + H_{2(g)} + Cl_{2(g)} + \frac{1}{2}O_{2(g)} \rightarrow Rb_2[UO_2Cl_4]\square 2H_2O$ **[-8.16 eV]**

ΔH₂: $\frac{1}{2}H_{2(g)} + \frac{1}{2}Cl_{2(g)} \rightarrow HCl_{(aq)}$ **[-1.73 eV]**

ΔH₃: $H_{2(g)} + \frac{1}{2}O_{2(g)} \rightarrow H_2O_{(l)}$ **[-2.96 eV]**

Overall: (ΔH₁ + -2ΔH₂ + -ΔH₃) $UO_{3(s)} + 2RbCl_{(s)} + 2HCl_{(aq)} + H_2O_{(l)} \rightarrow Rb_2[UO_2Cl_4]\square 2H_2O$ **[-1.74 eV]**

$Cs_2[UO_2Cl_4]$

ΔH₁: $UO_{3(s)} + 2CsCl_{(s)} + Cl_{2(g)} \rightarrow Cs_2[UO_2Cl_4] + \frac{1}{2}O_{2(g)}$ **[-0.92 eV]**

ΔH₂: $\frac{1}{2}H_{2(g)} + \frac{1}{2}Cl_{2(g)} \rightarrow HCl_{(aq)}$ **[-1.73 eV]**

ΔH₃: $H_{2(g)} + \frac{1}{2}O_{2(g)} \rightarrow H_2O_{(l)}$ **[-2.96 eV]**

Overall: (ΔH₁ + -2ΔH₂ + ΔH₃) $UO_{3(s)} + 2CsCl_{(s)} + 2HCl_{(aq)} \rightarrow Cs_2[UO_2Cl_4] + H_2O_{(l)}$ **[-0.42 eV]**

$(C_{10}H_{10}N_2)[UO_2Cl_4]$

ΔH₁: $UO_{3(s)} + C_{10}H_8N_{2(s)} + H_{2(g)} + 2Cl_{2(g)} \rightarrow (C_{10}H_{10}N_2)[UO_2Cl_4] + \frac{1}{2}O_{2(g)}$ **[-4.71 eV]**

ΔH₂: $\frac{1}{2}H_{2(g)} + \frac{1}{2}Cl_{2(g)} \rightarrow HCl_{(aq)}$ **[-1.73 eV]**

ΔH₃: $H_{2(g)} + \frac{1}{2}O_{2(g)} \rightarrow H_2O_{(l)}$ **[-2.96 eV]**

Overall: (ΔH₁ + -4ΔH₂ + ΔH₃) $UO_{3(s)} + C_{10}H_8N_{2(s)} + 4HCl_{(aq)} \rightarrow (C_{10}H_{10}N_2)[UO_2Cl_4] + H_2O_{(l)}$ **[-0.75 eV]**

$(C_{12}H_{12}N_2)[UO_2Cl_4]$

ΔH₁: $UO_{3(s)} + C_{12}H_{10}N_{2(s)} + H_{2(g)} + 2Cl_{2(g)} \rightarrow (C_{12}H_{12}N_2)[UO_2Cl_4] + \frac{1}{2}O_{2(g)}$ **[-4.96 eV]**

ΔH₂: $\frac{1}{2}H_{2(g)} + \frac{1}{2}Cl_{2(g)} \rightarrow HCl_{(aq)}$ **[-1.73 eV]**

ΔH$_3$: $H_{2(g)} + \frac{1}{2}O_{2(g)} \rightarrow H_2O_{(l)}$ **[-2.96 eV]**

Overall: (ΔH$_1$ + -4ΔH$_2$ + ΔH$_3$) $UO_{3(s)} + C_{12}H_{10}N_{2(s)} + 4HCl_{(aq)} \rightarrow (C_{12}H_{12}N_2)[UO_2Cl_4] + H_2O_{(l)}$ **[-0.99 eV]**

$(C_{12}H_{14}N_2)[UO_2Cl_4]$

ΔH$_1$: $UO_{3(s)} + C_{12}H_{12}N_{2(s)} + H_{2(g)} + 2Cl_{2(g)} \rightarrow (C_{12}H_{14}N_2)[UO_2Cl_4] + \frac{1}{2}O_{2(g)}$ **[-5.05 eV]**

ΔH$_2$: $\frac{1}{2}H_{2(g)} + \frac{1}{2}Cl_{2(g)} \rightarrow HCl_{(aq)}$ **[-1.73 eV]**

ΔH$_3$: $H_{2(g)} + \frac{1}{2}O_{2(g)} \rightarrow H_2O_{(l)}$ **[-2.96 eV]**

Overall: (ΔH$_1$ + -4ΔH$_2$ + ΔH$_3$) $UO_{3(s)} + C_{12}H_{14}N_{2(s)} + 4HCl_{(aq)} \rightarrow (C_{12}H_{14}N_2)[UO_2Cl_4] + H_2O_{(l)}$ **[-1.08 eV]**

S6.2 Formation Enthalpies Referenced to Standard States

Table S19: Calculated formation enthalpies of the uranyl-cation and uranyl-hydrogen structures as referenced to the standard state of each atom.

Uranyl Structure	Formation Enthalpy Formula	Calculated $\Delta H°_f$ (kJ/mol)
**(1)**	$U_{(s)} + 2K_{(s)} + 2H_{2(g)} + 2Cl_{2(g)} + 2O_{2(g)} \rightarrow K_2[UO_2Cl_4]$□$2H_2O$	-2583.27
**(2)**	$U_{(s)} + 2Rb_{(s)} + 2H_{2(g)} + 2Cl_{2(g)} + 2O_{2(g)} \rightarrow Rb_2[UO_2Cl_4]$□$2H_2O$	-2587.27
**(3)**	$U_{(s)} + 2Cs_{(s)} + 2Cl_{2(g)} + O_{2(g)} \rightarrow Cs_2[UO_2Cl_4]$	-2062.00
**(4)**	$U_{(s)} + 10C_{(s)} + 5H_{2(g)} + N_{2(g)} + 2Cl_{2(g)} + O_{2(g)} \rightarrow (C_{10}H_{10}N_2)[UO_2Cl_4]$	-1441.29
**(5)**	$U_{(s)} + 12C_{(s)} + 6H_{2(g)} + N_{2(g)} + 2Cl_{2(g)} + O_{2(g)} \rightarrow (C_{12}H_{12}N_2)[UO_2Cl_4]$	-1519.93
**(6)**	$U_{(s)} + 12C_{(s)} + 7H_{2(g)} + N_{2(g)} + 2Cl_{2(g)} + O_{2(g)} \rightarrow (C_{12}H_{14}N_2)[UO_2Cl_4]$	-1425.00

S7. Cohesive Energies

Cohesive energies for the ionic crystals KCl, RbCl, and CsCl were calculated using a thermodynamic cycle as shown below.

KCl

1) $KCl_{(s)} \rightarrow K_{(g)} + Cl_{(g)}$
2) $K_{(g)} \rightarrow K^{+}_{(g)} + e^{-}$
3) $Cl_{(g)} + e^{-} \rightarrow Cl^{-}_{(g)}$

Overall: $KCl_{(s)} \rightarrow K^{+}_{(g)} + Cl^{-}_{(g)}$

RbCl

1) $RbCl_{(s)} \rightarrow Rb_{(g)} + Cl_{(g)}$
2) $Rb_{(g)} \rightarrow Rb^{+}_{(g)} + e^{-}$
3) $Cl_{(g)} + e^{-} \rightarrow Cl^{-}_{(g)}$

Overall: $RbCl_{(s)} \rightarrow Rb^{+}_{(g)} + Cl^{-}_{(g)}$

CsCl

1) $CsCl_{(s)} \rightarrow Cs_{(g)} + Cl_{(g)}$
2) $Cs_{(g)} \rightarrow Cs^{+}_{(g)} + e^{-}$
3) $Cl_{(g)} + e^{-} \rightarrow Cl^{-}_{(g)}$

Overall: $CsCl_{(s)} \rightarrow Cs^{+}_{(g)} + Cl^{-}_{(g)}$

In steps (2) and (3), the energies for alkali metal ionization potentials and the chlorine electron affinity were taken from Ref [11].

Cohesive energies of the organic crystals were calculated using:

$$\frac{nE_{molecule} - E_{solid}}{n}$$

where $E_{molecule}$ represents the isolated organic molecule in the gas phase, E_{solid} represents the organic crystal, and n corresponds to the total number of molecules in the unit cell.

Table S20. Calculated cohesive energies for the ionic and organic reactant crystals.

Ionic System	Cohesive Energy (kJ/mol*atom)
**KCl**	360.30
**RbCl**	345.50
**CsCl**	328.73
**Organic System**	**Cohesive Energy (kJ/mol*molecule)**
**$C_{10}H_8N_2$**	101.74
**$C_{12}H_{10}N_2$**	122.87
**$C_{12}H_{12}N_2$**	120.37

S8. DFT Optimized Structures

Below are the final CONTCAR files from the optimized PBE+U+D3-BJ structures.

(1) $K_2[UO_2Cl_4]$□$2H_2O$

```
     6.6712455248917930   -0.0552695280299233    0.0536682142844467
    -3.2895728389579904    5.8807669862593119    0.0049466675188942
    -1.4313165447360898   -1.1176478023868619    7.0049288200175344
   Cl   K    O    H    U
     4     2     4     4     1
Direct
  0.2925437779289766  0.2659189503916366  0.1430581532157973
  0.7074561550710214  0.7340810516083636  0.8569418507842030
  0.3176385712286773  0.0981425899832331  0.6400172508413178
  0.6823614657713222  0.9018574620167641  0.3599827531586826
  0.9262677139426145  0.2559706337408514  0.7623902464543804
  0.0737322180573869  0.7440293542591476  0.2376097405456221
  0.7596068990435114  0.4881205335548628  0.4942548582454904
  0.2403930759564901  0.5118794744451378  0.5057450787545150
  0.7625541209422337  0.2838504187241426  0.0750923249767723
  0.2374458630577720  0.7161496152758531  0.9249076370232316
  0.2355811565226347  0.8243694182796162  0.8388624355830814
  0.7644187874773678  0.1756305667203861  0.1611375354169198
  0.3939956105483517  0.7323927842231228  0.9342987717145377
  0.6060043704516502  0.2676072327768750  0.0657012112854645
  0.4999999979999998  0.5000000280000023  0.4999999850000023
```

(2) $Rb_2[UO_2Cl_4]$□$2H_2O$

```
     6.8029859797970413   -0.0678345835440302    0.0607070256830652
    -3.4031806400312545    6.0765840553773351    0.0074195011537665
    -1.5058937090570257   -1.0974387787679145    7.1905456973771820
   O    Cl   Rb   H    U
     4     4     2     4     1
Direct
  0.2576589795006257  0.9935621758532847  0.9971012006071405
  0.7423410474993730  0.0064378031467172  0.0028987993928595
  0.7447457062887182  0.2303687538030346  0.4137594392860393
  0.2552542957112820  0.7696312441969653  0.5862405297139617
  0.1956951598342656  0.2226748730748938  0.3490536005303966
  0.8043048301657407  0.7773251209250986  0.6509464004696000
  0.1746752519811778  0.3896170522406806  0.8606681987867049
  0.8253247300188278  0.6103828477593183  0.1393318192132966
  0.5739046016115665  0.2473443719682180  0.7358371199243976
  0.4260954233884249  0.7526555870317821  0.2641628980756039
  0.8959033324751360  0.2419785095316840  0.4194384085078582
  0.1040966225248638  0.7580215064683173  0.5805616264921412
  0.7428769149211689  0.3356635142231212  0.3298665057576855
  0.2571230110788321  0.6643364207768769  0.6701334962423076
  0.0000000000000000  0.0000000000000000  0.0000000000000000
```

(3) $Cs_2[UO_2Cl_4]$

```
    11.9571179280429760    0.0000000000000000    0.0302949746630793
     0.0000000000000000    7.7815324961761041    0.0000000000000000
    -1.0387938907089320    0.0000000000000000    5.7207942428008263
   U    Cs   Cl   O
     2     4     8     4
Direct
  0.0000000000000000  0.0000000000000000  0.0000000000000000
  0.5000000000000000  0.5000000000000000  0.0000000000000000
  0.6651462089592712  0.0000000000000000  0.3194072487280835
  0.3348537660407302  0.0000000000000000  0.6805927132719205
  0.1651462189592721  0.5000000000000000  0.3194072487280835
  0.8348537460407286  0.5000000000000000  0.6805927132719205
  0.8978635194619358  0.7476998811153948  0.2070391628060690
  0.1021364105380655  0.2523001338846100  0.7929608611939329
  0.1021364105380655  0.7476998811153948  0.7929608611939329
  0.8978635194619358  0.2523001338846100  0.2070391628060690
  0.3978635194619358  0.2476998191153896  0.2070391628060690
  0.6021364005380647  0.7523001188846052  0.7929608611939329
  0.6021364005380647  0.2476998191153896  0.7929608611939329
  0.3978635194619358  0.7523001188846052  0.2070391628060690
  0.1170252085425787  0.0000000000000000  0.2357747100754395
  0.8829748324574211  0.0000000000000000  0.7642253139245625
  0.6170252085425787  0.5000000000000000  0.2357747100754395
  0.3829748124574195  0.5000000000000000  0.7642253139245625
```

(4) $C_{10}H_{10}N_2[UO_2Cl_4]$

```
     5.4781233519013837    0.0167618769110367    0.0473955311188962
     0.5361833004956608    8.5020006773797494    0.0639743972680069
     2.6063690020290622    2.7528569139702337    8.0349965881460754
   U    Cl    O     N     H     C
     1     4     2     2    10    10
Direct
  -0.0000000000000000  0.0000000000000000  0.0000000000000000
   0.1680204409580948  0.3109565841880038  0.9463697925854218
   0.8319795570419047  0.6890433848119973  0.0536302064145745
   0.0154496887511588  0.9241571076318279  0.3182682372384383
   0.9845502742488417  0.0758428353681713  0.6817318437615582
   0.6826687708707340  0.0650749487243544  0.0652149645431896
   0.3173311971292705  0.9349250062756457  0.9347851014568089
   0.3733668924516982  0.2917733221506739  0.2511389854808664
   0.6266330665483020  0.7082266488493203  0.7488610955191372
   0.3405661803897959  0.2351996824344007  0.1723341372409527
   0.6594338866101991  0.7648002745655993  0.8276658987590503
   0.6168183868405738  0.1071126133981650  0.3331382457932435
   0.3831816191594192  0.8928873026018349  0.6668617452067596
   0.7085340952981398  0.2417622312199149  0.5187463136942605
   0.2914658797018616  0.7582377217800850  0.4812537373057402
   0.2093307095598216  0.6372559692099022  0.3476820855173008
   0.7906691924401771  0.3627439927900944  0.6523179364827009
   0.1338684711360184  0.4813893759933187  0.1734779112562414
   0.8661315188639810  0.5186105720066844  0.8265221547437571
   0.5308694856821977  0.2231157808925778  0.3440535374894754
   0.4691305553178018  0.7768842181074258  0.6559464545105245
   0.5812389662409225  0.3024071370433550  0.4447935057755346
   0.4187609777590797  0.6975928529566441  0.5552066052244715
   0.4698559550462748  0.4547650028781287  0.4493517919082292
   0.5301440459537287  0.5452349691218686  0.5506482000917700
   0.3017710112740896  0.5191649967969902  0.3527792562198949
   0.6982290057259085  0.4808349562030095  0.6472207947801056
   0.2565957814744382  0.4353244024740676  0.2542397621771754
   0.7434042915255574  0.5646755595259292  0.7457602588228229
```

(5) $C_{12}H_{12}N_2[UO_2Cl_4]$

```
      6.9664285996033106    0.0534499224258788   -0.0276841198442231
      3.2627673829656452    7.4731846967735800    0.0419239678127975
      2.3053241466998089    0.4112118818963590    7.9805963782954574
   U    Cl    O     N     H     C
     1     4     2     2    12    12
Direct
 -0.0000000000000000  0.0000000000000000 -0.0000000000000000
  0.7645709686327524  0.9585422174489134  0.3086990567897535
  0.2354289993672450  0.0414578025510884  0.6913009812102567
  0.1543222495696579  0.2051838737311630  0.1126754388857981
  0.8456777164303393  0.7948161292688409  0.8873246291142004
  0.7796669020680008  0.1982875811614574  0.9626072701469749
  0.2203330769320009  0.8017124068385415  0.0373927788530255
  0.4398640137688040  0.3117505932830647  0.7595391929704293
  0.5601360422312003  0.6882493627169322  0.2404608750295762
  0.3325874096747176  0.2511933365040248  0.8177857837360426
  0.6674126893252869  0.7488065574959736  0.1822142402639591
  0.6146571860368816  0.0999599314968181  0.6032724763413299
  0.3853427909631200  0.9000400895031802  0.3967275316586708
  0.2665414741477964  0.5295966890758094  0.9096387842614726
  0.7334585188522064  0.4704032659241902  0.0903612687385279
  0.8945637218743487  0.2265862386120572  0.4617932002803813
  0.1054361951256479  0.7734137673879434  0.5382068077196193
  0.5325913628143130  0.6767988009085284  0.7824001698756265
  0.4674086151856922  0.3232011870914705  0.2175998831243743
  0.8536281509282525  0.6767255456631874  0.5880860092646358
  0.1463717980717470  0.3232744433368151  0.4119140287353603
  0.6091720853606686  0.2251639262117987  0.6373609295361193
  0.3908279116393348  0.7748360307882014  0.3626391084638766
  0.4096832827139614  0.4703342632251890  0.8117499316322724
  0.5903167002860410  0.5296657257748135  0.1882501053677270
  0.7618711378995199  0.2975949973983278  0.5620796001127522
  0.2381288531004755  0.7024049916016748  0.4379204078872415
  0.5580178889023183  0.5485020885701051  0.7399228941285970
  0.4419820730976787  0.4514979644298956  0.2600771138714032
  0.7416414794409255  0.4608627707201540  0.6152622849207861
  0.2583584355590779  0.5391372172798450  0.3847377520792204
  0.9011285071930377  0.5414349817736485  0.5494258559691383
  0.0988714748069608  0.4585650062263509  0.4505741820308652
```

(6) $C_{12}H_{14}N_2[UO_2Cl_4]$

```
     7.0592117595936195    0.0502751684405794   -0.0213403194817532
     3.4126334546670791    7.5561070252922722    0.0993276913469466
     2.8925559838962633    0.0509146655615266    8.0318559785578945
   U    Cl   O    N    H    C
     1     4     2     2    14    12
Direct
  0.4999999850000023 -0.0000000000000000  0.4999999870000025
  0.2348145928315437  0.9837664607149966  0.8060968946825585
  0.7651853631684596  0.0162335482850076  0.1939030493174440
  0.3404578662743732  0.8065338350260880  0.3878098316069670
  0.6595422647256269  0.1934662339739070  0.6121901133930320
  0.2937282291531469  0.2021079076252706  0.4610302167345017
  0.7062717728468533  0.7978920993747335  0.5389697852654983
  0.9662294040100872  0.2950137228385603  0.2816697714455529
  0.0337706169899182  0.7049862681614354  0.7183302015544414
  0.8652434540479856  0.2374883322099262  0.3642749706920119
  0.1347565559520080  0.7625117057900699  0.6357249733079835
  0.1393756879809875  0.0695428926488371  0.1380715686115543
  0.8606242830190136  0.9304571143511742  0.8619283823884453
  0.4011382767557514  0.1984086262447092  0.9427605271663505
  0.5988617232442486  0.8015913957552926  0.0572394598336520
  0.7965099673359267  0.5247366652309423  0.4207297185830181
  0.2034899946640702  0.4752633567690596  0.5792701974169820
  0.0414602945655300  0.6733694914235085  0.2381683047417009
  0.9585397794344761  0.3266305315764897  0.7618316682582933
  0.3187209676635377  0.6697903790692303  0.9602702882333376
  0.6812789693364607  0.3302096749307671  0.0397296517666575
  0.4627474058543758  0.4736379059480655  0.8402515829662284
  0.5372525511456242  0.5263621160519292  0.1597483470337586
  0.1300243003941545  0.2005193758107893  0.1531426722114987
  0.8699757316058410  0.7994806301892112  0.8468572577885026
  0.2717986417613544  0.2739557246105526  0.0484818701292125
  0.7282014582386468  0.7260442973894493  0.9515181208707902
  0.9337077310200285  0.4601190321899365  0.3139378570680835
  0.0662923579799682  0.5398809588100663  0.6860621159319179
  0.0717889813453811  0.5386406580532670  0.2124607244734492
  0.9282109846546232  0.4613593639467277  0.7875392625265462
  0.2453240745073962  0.4452915704585909  0.0769655905418194
  0.7546759254926039  0.5547084525414074  0.9230343964581830
  0.4014070649753990  0.5245708263259006  0.9668430276775202
  0.5985930130246073  0.4754292276740968  0.0331569003224810
```

S9. References

1. Enriquez, E.; Wang, G.; Sharma, Y.; Sarpkaya, I.; Wang, Q.; Chen, D.; Winner, N.; Guo, X.; Dunwoody, J.; White, J.; Nelson, A.; Xu, H.; Dowden, P.; Batista, E.; Htoon, H.; Yang, P.; Jia, Q.; Chen, A., Structural and Optical Properties of Phase-Pure UO2, α-U3O8, and α-UO3 Epitaxial Thin Films Grown by Pulsed Laser Deposition. *ACS Applied Materials & Interfaces* **2020,** *12* (31), 35232-35241.
2. Boag, N. M.; Coward, K. M.; Jones, A. C.; Pemble, M. E.; Thompson, J. R., 4,4'-Bipyridyl at 203K. *Acta Crystallographica Section C* **1999,** *55* (4), 672-674.
3. Ide, S.; Karacan, N.; Tufan, Y., 1,2-Bis(4-pyridyl)ethane. *Acta Crystallographica Section C* **1995,** *51* (11), 2304-2305.
4. Schnaars, D. D.; Wilson, R. E., Structural and Vibrational Properties of U(VI)O2Cl42- and Pu(VI)O2Cl42- Complexes. *Inorganic Chemistry* **2013,** *52* (24), 14138-14147.
5. Deifel, N. P.; Cahill, C. L., The uranyl tetrachloride anion as a tecton in the assembly of U(VI) hybrid materials. *CrystEngComm* **2009,** *11* (12), 2739-2744.
6. Donald D. Wagman, W. H. E., Vivian B. Parker, Richard H. Schumm, Iva Halow, Sylvia M. Bailey, Kenneth L. Churney, Ralph L. Nuttall, The NBS Tables of Chemical Thermodynamic Properties: Selected values for inorganic and C1 and C2 organic substances. *Journal of Physical and Chemical Reference Data* **1982,** *11*.
7. Abbaspour Tamijani, A.; Augustine, L. J.; Bjorklund, J. L.; Catalano, J. G.; Mason, S. E., First-Principles Characterisation and Comparison of Clean, Hydrated, and Defect Al2O3 and Fe2O3 (110) Surfaces. *Under Review* **2021**.
8. Augustine, L. J.; Abbaspour Tamijani, A.; Bjorklund, J. L.; Al-Abadleh, H. A.; Mason, S. E., Adsorption of small organic acids and polyphenols on hematite surfaces: Density Functional Theory + thermodynamics analysis. *Journal of Colloid and Interface Science* **2022,** *609*, 469-481.
9. Tamijani, A. A.; Bjorklund, J. L.; Augustine, L. J.; Catalano, J. G.; Mason, S. E., Density Functional Theory and Thermodynamics Modeling of Inner-Sphere Oxyanion Adsorption on the Hydroxylated α-Al2O3(001) Surface. *Langmuir* **2020,** *36* (44), 13166-13180.
10. Mason, S. E.; Iceman, C. R.; Trainor, T. P.; Chaka, A. M., Density functional theory study of clean, hydrated, and defective alumina $(1\overline{1}02)$ surfaces. *Physical Review B* **2010,** *81* (12), 125423.
11. Kittel, C., *Introduction to solid state physics*. 8th Edition ed.; Wiley: New York, 1966.
